# Towards a comprehensive framework for movement and distortion correction of diffusion MR images: Within volume movement

**DOI:** 10.1016/j.neuroimage.2017.02.085

**Published:** 2017-05-15

**Authors:** Jesper L.R. Andersson, Mark S. Graham, Ivana Drobnjak, Hui Zhang, Nicola Filippini, Matteo Bastiani

**Affiliations:** a FMRIB Centre, Oxford University, Oxford, United Kingdom; b Centre for Medical Image Computing & Department of Computer Science, University College London, London, United Kingdom; c Department of Psychiatry, Oxford University, Oxford, United Kingdom

**Keywords:** Diffusion, Movement, Slice-to-volume, Registration, Interpolation

## Abstract

Most motion correction methods work by aligning a set of volumes together, or to a volume that represents a reference location. These are based on an implicit assumption that the subject remains motionless during the several seconds it takes to acquire all slices in a volume, and that any movement occurs in the brief moment between acquiring the last slice of one volume and the first slice of the next. This is clearly an approximation that can be more or less good depending on how long it takes to acquire one volume and in how rapidly the subject moves. In this paper we present a method that increases the temporal resolution of the motion correction by modelling movement as a piecewise continous function over time. This intra-volume movement correction is implemented within a previously presented framework that simultaneously estimates distortions, movement and movement-induced signal dropout. We validate the method on highly realistic simulated data containing all of these effects. It is demonstrated that we can estimate the true movement with high accuracy, and that scalar parameters derived from the data, such as fractional anisotropy, are estimated with greater fidelity when data has been corrected for intra-volume movement. Importantly, we also show that the difference in fidelity between data affected by different amounts of movement is much reduced when taking intra-volume movement into account. Additional validation was performed on data from a healthy volunteer scanned when lying still and when performing deliberate movements. We show an increased correspondence between the “still” and the “movement” data when the latter is corrected for intra-volume movement. Finally we demonstrate a big reduction in the telltale signs of intra-volume movement in data acquired on elderly subjects.

## Highlights

• We introduce a method to correct for intra-volume movement into an existing framework for movement and distortion correction.
• It has been validated on realistic simulated data and on experimental data with deliberate movement.
• The results indicate that one can reliably reverse the adverse effects of intra-volume movement.

## 1 Introduction

In previous work (Andersson and Sotiropoulos, 2015, Andersson and Sotiropoulos, 2016, Andersson et al., 2016) we have described a framework for the simultaneous correction of susceptibility- and eddy current-induced distortions as well as gross movement and signal loss caused by subject movement. We have argued for incorporating all of these effects into a comprehensive model since this is how they originate, and any attempt at a sequential correction strategy is bound to be inferior. In the present paper we augment that framework with a model for within volume movement (Kim et al., 1999). We will refer to the software that implements the framework described in this paper as eddy.

Note that there are two distinct artefects in the data that are both associated with intra-volume movement

**Signal loss.**

This is an artefact that can occur when a movement coincides exactly in time with the diffusion encoding part of the sequence. It can occur even for very small movement, such that if the signal had been present it would have sampled the correct slice in space.

**Slices irregularly sampled in space.**

When movement occurs between the acquistion of slices within a volume (but not exactly coinciding with the diffusion encoding) the slices will be acquired “out of order”. One can think of the stack of slices as a deck of cards, and of the effect of intra-volume movement as these cards no longer being stacked perfectly on top of each other. In our case, unlike that of a deck of cards, out-of-plane movement can also occur in which these “cards” can also intersect each other, swap places etc.

The former of these effects were the topic of a previous paper (Andersson et al., 2016). This work is concerned only with the latter effect.

A common implicit assumption in motion correction is that the subject remains motionless during the acquisition of all the slices that when stacked together constitute an image volume. Any movement is assumed to occur in the milliseconds between the acquisition of the last slice in one volume and the start of the acquisition of the first slice in the next. Of course no one believes that, but it is a reasonable assumption that movement is continous and slow over time. This would mean that, on average, movement within a volume is small compared to movement that has occurred between volumes that were acquired some number of repetition-times apart. The popularity and relative success of the many methods based on that assumption (Jiang et al., 1995, Friston et al., 1995, Jenkinson et al., 2002) shows that it has some merit. On the other hand there is strong evidence that volumetric (meaning volume-to-volume) realignment does not completely correct for all movement induced effects (Friston et al., 1996) and that part of this residual variance comes from within volume movement (Beall and Lowe, 2014). For the case of BOLD imaging this has lead to various strategies for removing offending volumes (Power et al., 2014) or regressing out selected variance components (Salimi-Khorshidi et al., 2014).

When a subject moves during the acquisition of the slices constituting a volume, the resulting 3D brain volume will be distorted relative to its true shape. In diffusion imaging slices are usually acquired in an interleaved order in order to minimise cross-talk, in which case the distortions will cause tell-tale jagged edges when volumes are viewed as coronal and/or sagittal slices (see Fig. 6 for an example). These relatively rapid movements are a more frequent problem with data from less coorporative subjects such as infants, children and subjects with dementia. Because of the logistics associated with scanning these groups, the data from them are particularly valuable and it is not an attractive prospect to have to remove volumes because of within volume movement.

This paper presents a new method for slice-to-volume registration that has been integrated in a previously presented framework for the simultaneous correction of distortions, subject movement (Andersson and Sotiropoulos, 2016) and movement-induced signal loss (Andersson et al., 2016). It is based on a continuous movement model, where each movement parameter (*x*-, *y*- and *z*-translation and rotation around the *x*-, *y*- and *z*-axes) is modeled using a discrete cosine (DCT) basis set. The (movement) parameters of the DCT model are estimated by comparing predicted with observed diffusion weighted slices. The reverse problem, to resample the irregularly acquired slices onto a regular grid, is solved by a novel hybrid 2D+1D resampling strategy. In brief, assuming axial slices, each acquired slice is spline interpolated onto a regular *xy*-grid in the target space, followed by irregular 1D spline resampling along the resulting *z*-columns. We show, using highly realistic simulated data, that it is able to accurately estimate movement parameters with a temporal resolution much greater than the one dictated by repetition time. We further demonstrate that for simulated data affected by intra-volume movement, the suggested method yields fractional anisotropy (FA) values closer to the truth, and also that there is significantly less difference in FA between data acquired with different amounts of movement. Finally, we show an example of how the method performs on a real dataset with severe movement.

## 2 Theory

### 2.1 Generative model for diffusion data

At the heart of the framework is a generative model used to make predictions about diffusion data (Andersson and Sotiropoulos, 2015, Andersson and Sotiropoulos, 2016). The model is based on a Gaussian Process (Rasmussen and Williams, 2006) where the hyperparameters are estimated from the data at hand (Andersson and Sotiropoulos, 2015). It is effectively an interpolating filter in Q-space, and for the early iterations, also in physical space, since data for a given voxel location is likely to originate from many different physical locations because of EC-induced distortions and subject movement. It is helpful to conceptualise it as a “Prediction Maker” (PM) that takes as input a set of image data-diffusion parameter pairs $F=\left\{\{f_{i},d_{i}\}:i\in N\right\}$ where *f*_*i*_ is the *i*th image volume, where $d_{i}$ describes the direction and magnitude of the diffusion weighting applied when acquiring *f*_*i*_ and where *N* is the total number of image volumes in the data set. Given input F and a diffusion descriptor $d_{*}$ (where * denotes any possible direction, not restricted to *N*), PM will return a prediction of what a corresponding image volume f^* would look like, i.e.

(1) f^*=PM(d*:F)

If the images in the set F are differentially distorted and/or affected by subject movement, the output f^* will be at a point in location-distortion space that represents an average of the images in F, weighted towards the images $\left\{f_{i}\right\}$ whose diffusion descriptors $\left\{d_{i}\right\}$ are close to $d_{*}$. f^* will also be “spatially smoothed” by the range of movement/distortion in the images *f*_*i*_ in the vicinity of $d_{*}$.

If, on the other hand, the images in F are all at the same point in movement/distortion space the image f^* will be at that same point, and there will be no “spatial smoothing”. In that case f^* represents only a Q-space interpolation/smoothing of the input data F.

### 2.2 Temporal subject movement model

The rigid body model is typically parametrised by three translations and three rotations $r=[\Delta_{x}\;\Delta_{y}\;\Delta_{z}\;\phi_{x}\;\phi_{y}\;\phi_{z}]$ from which a one-augmented transformation matrix R(r) is created such that

(2) $$\left[\begin{array}{c} x\prime \\ 1 \end{array}\right]=R(r)\left[\begin{array}{c} x\\ 1 \end{array}\right]$$

where x is a coordinate in the target space, and x′ is a coordinate in the space of an image one wants to resample.

For volumetric registration a single $r_{i}$ is estimated for each image volume *f*_*i*_ and the resulting $R(r_{i})$ is applied to all coordinates x on a regular grid in a target volume. When extending this to a within volume movement model, we still want to retain the assumption that movement is continuous over time. Hence we would like to have a smooth function $r(t)=[\Delta_{x}(t)\;\Delta_{y}(t)\;\Delta_{z}(t)\;\phi_{x}(t)\;\phi_{y}(t)\;\phi_{z}(t)]$. We have chosen to model this with a discrete cosine transform (DCT) basis set such that

(3) $$r(t_{n})=\left[\begin{array}{ccccc} 1 & \cos\frac{n\pi }{N-1} & \cos\frac{2n\pi }{N-1} & \cdots & \cos\frac{\mathrm{Mn}\pi }{N-1} \end{array}\right]B$$

where it is assumed that the volume was acquired at *N* discrete time-points (these would correspond to slices or multi-band groups) and where *n* can be any discrete time point between 0 and N−1. *M* is the order of the expansion and can be any integer not larger than N−1. The M×6 matrix B consists of

(4) $$B=\left[\begin{array}{cccc} \Delta_{x}^{\left(0\right)} & \Delta_{y}^{\left(0\right)} & \cdots & \phi_{z}^{\left(0\right)}\\ \Delta_{x}^{\left(1\right)} & \Delta_{y}^{\left(1\right)} & \cdots & \phi_{z}^{\left(1\right)}\\ \vdots & \vdots & \ddots & \vdots \\ \Delta_{x}^{\left(M\right)} & \Delta_{y}^{\left(M\right)} & \cdots & \phi_{z}^{\left(M\right)} \end{array}\right]$$

and is the set of unknown parameters that need to be estimated for each volume for the within volume movement model. Note that if *M*=0, $r(t_{n})$ becomes $\left[\Delta_{x}^{\left(0\right)}\;\Delta_{y}^{\left(0\right)}\;\Delta_{z}^{\left(0\right)}\;\phi_{x}^{\left(0\right)}\;\phi_{y}^{\left(0\right)}\;\phi_{z}^{\left(0\right)}\right]$, i.e. it reverts to a model assuming no intra-volume movement.

This is then combined with a function $Z(t_{n})$ that returns all the slice indices that were acquired at time *t*_*n*_. In the simplest case of single-slice sequential acquisition Z(0)=0, Z(1)=1 etc, and for a single slice interleaved acquisition Z(0)=0, Z(1)=2,…,Z(N/2)=1, Z(N/2+1)=3 etc. For a slightly more involved case with *N*=30, a multi-band (MB) factor of 3 and interleaved acquisition the slices are acquired in groups (MB-groups) where Z(0)=[01020], Z(1)=[21222] etc.

This leads us to the following algorithm for a single volume of the intra-volume movement model where we assume that B is known 

#### 2.2.1 Regularisation of movement over time

The DCT basis-set described above guarantees that the estimated movement trace is continuous and differentiable, but it does not put any limits on speed or acceleration of movement. Our initial tests showed that when combined with an eddy current distortion model this leads to a poorly conditioned matrix for the Gauss-Newton step that is used to update the movement and distortion parameters. Therefore we also include a regularisation term on the movement. It penalises the integral of the second derivatives of movement w.r.t. time, calculated by taking the analytical derivative at each time point (slice or MB-group) and summing over all time points. The regularisation term is weighted by an arbitrary constant *λ*, where a small *λ* yields movement traces that vary more rapidly over time and a large *λ* yields traces that vary more slowly. Through empirical testing we found that *λ* in the range 0.1<λ<100 yields good results. In the paper we demonstrate results for λ=1 and λ=10.

### 2.3 Forward spatial model

The purpose of the forward spatial model is to predict what an observed image *f*_*i*_ would look like if during its acquisition it had been affected by movement B, an eddy current-induced field e(β) and a susceptibility induced field *h*. It starts with an image volume f^i calculated from Eq. (1). This prediction will be “complete” in the sense that it exists for all voxels of the regular grid that it is defined on. That prediction will be transformed into slice *s* in observation space by sampling it at coordinates given by

(5) $$x\prime =R(r_{i}(s))\left(x+d_{x}(\omega (h,r_{i}(s))+e{\left(\beta_{i}),a_{i}\right)}^{-1}\right)$$

where $r_{i}(s)$ denotes the movement parameters for slice *s* of volume *i* and where the other symbols are explained in appendix Appendix A. This model is the same as the one introduced in Andersson and Sotiropoulos (2016), extended to intra-volume movement. Importantly, by the nature of their estimation, both *h* and e(β) are defined for all points in our model space so it is straightforward to calculate the transform given by Eq. (5) for all slices *s*. Note though that if one was to stack the different $\omega (h,r_{i}(s))$ for all slices *s* and view them as a volume it would be “disjointed”, because the underlying susceptibility-induced field *h* has been sampled in an irregular fashion .

**Fig. 1:**
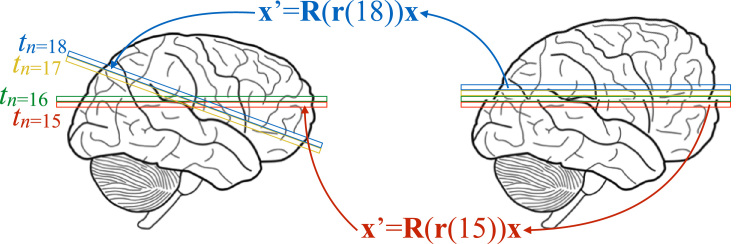
This figure serves the double purpose of explaining the intra-volume movement problem as well as our forward spatial model. To understand the problem, consider a sequential single-slice sequence where slices 15 and 16 have just been acquired, then the subjects move (“looking up into the sky”) so that slices 17 and 18 are acquired at the positions indicated in the schematic on the left. The reconstruction process does not know that the subject has moved and will stack the slices on top of each other as seen on the right hand side. If we assume that the subject stays in this new position for the remaining slices the apparent shape of the brain will now be as seen on the right (looking more like a sperm whale than a brain), as opposed to the true shape shown on the left. In order to understand the forward model, assume that all movement is known such that we can accurately calculate the matrices R(r(15)) and R(r(18)). The image f^i from Eq. (1) serves as the image on the left, and is hence “known”. The aim of the forward model is now to calculate the image on the right given that we know B, i.e. all the movements. This is performed using the following strategy: for all coordinates x on the right calculate x′, map x′ into the regular grid of f^i and use standard spline interpolation to calculate an intensity f^i(x′) that is written into $f_{i}(x)$ on the right. Using this strategy it is possible to predict the “observed” image for any set of movements B.

### 2.4 Inverse spatial model

**Fig. 2:**
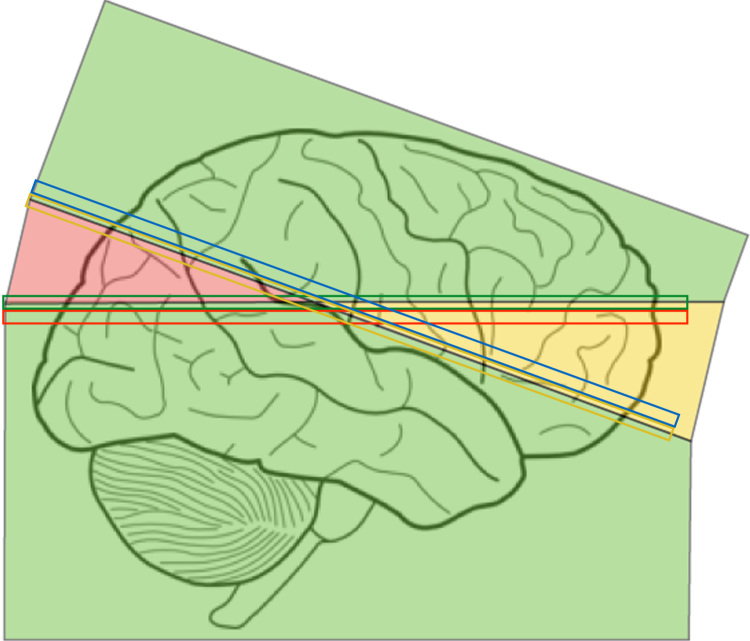
This figure shows the same acquisition situation as Fig. 1. After acquisition of slices 15 (red slice) and 16 (green) the subject moved (“looked into the sky”) and slices 17 and 18 were acquired in the locations shown in yellow and blue respectively. We also assume that prior to slice 15 all slices were acquired with the subject in the same position and hence were parallel to slice 15 and correspondingly all slices after slice 18 were parallel to slice 18. The green parts of the volume have been acquired once, and once only, on a regular grid and standard interpolation would in principle be feasible. The yellow part has been acquired twice and there will be spots where voxel values from one slice will almost exactly coincide (spatially) with values from another slice and these values will have to be reconciled. Finally the red part has not been acquired at all which means that an interpolation will need to fill in values a long distance from the nearest observed values. For an interleaved acquisition, which is more typical, one would instead have multiple smaller yellow and red areas in close proximity, but the principle is the same.

This is a considerably more difficult problem than the “Forward spatial model” described above in Section 2.3. When there is movement during the acquisition of the slices constituting a volume the samples will not be on a regular grid, which complicates the use of standard interpolation algorithms (see for example (Lee et al., 1997)). If the movements are out-of-plane, there is also a risk that there are gaps in the volume where no data has been acquired. The problem is explained graphically in Fig. 2.

In order to make the explanation clearer we simplify the model to only consider slice-wise movement, i.e. we have ignored e(β) and *h*. The full equations, including e(β) and *h*, are given in appendix Appendix A. For now we will only note that both e(β) and *h* exist for all voxels.

**Fig. 3:**
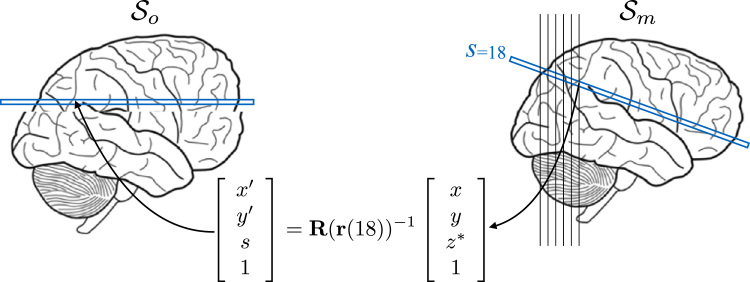
Again, this figure uses the same acquisition situation as in Fig. 1. We wish to use the slices in $S_{o}$ to re-create a volume in $S_{m}$. In order to do so we need to know what x′ and y′ coordinates in the original slice *s* in $S_{o}$ correspond to integer/regular coordinates *x* and *y* in $S_{m}$. We also need to know what non-integer coordinate $z^{*}$ in $S_{m}$ corresponds to every combination of *x*, *y* and *s*. That leads to the equation in the figure, where there are unknowns on both sides (x′, y′ in $S_{o}$ and $z^{*}$ in $S_{m}$).

We have approached the problem by dividing it into a 2D interpolation on a regular grid, followed by a 1D irregular interpolation. Let us say that we have two spaces: $S_{m}$, which is the space that we want to interpolate into, and $S_{o}$, which is the space of the image that has been acquired. Note that $S_{m}$ is defined by a regular grid and that $S_{o}$ is the space where the slices are potentially irregularly organised. Consider a column in the space $S_{m}$, defined by [xy*], i.e. an *x* and a *y* coordinate and where the * denotes “any *z*-value”. Each slice in $S_{o}$ is acquired on a regular 2D grid, so for any slice *s* we can find an x′ and a y′ corresponding to that [xy] pair, and we can get a 2D interpolated value for that x′ and y′. We can also for that [x′y′] pair calculate a $z^{*}$ coordinate that tells us the *z*-coordinate in $S_{m}$ that this 2D interpolated value corresponds to. This yields a set of columns in $S_{m}$, one for each [xy] pair, with 2D interpolated intensity values *g*_*i*_ and a $z_{i}^{*}$ coordinate associated with each value. We have illustrated this process in Fig. 3.

The equation

(6) $$\left[\begin{array}{c} x\prime \\ y\prime \\ s\\ 1 \end{array}\right]=R{\left(r(s)\right)}^{-1}\left[\begin{array}{c} x\\ y\\ z^{*}\\ 1 \end{array}\right]$$

in Fig. 3 is different from the “normal” transform (for example Eq. (2)) in that the unknowns (x′, y′ and $z^{*}$) appear on both sides of the equals sign. The coordinates x′ and y′ define the point to be sampled in slice *s* in the observed volume ($S_{o}$), but *s* is given a-priori and is hence known. *x* and *y* correspond to the regular sampling points in $S_{m}$, but $z^{*}$ is given by the plane that is defined by *s* and $R^{-1}$. By writing out the individual equations of Eq. (6) it becomes obvious how to solve it

(7a) $$x\prime =R_{11}^{I}x+R_{12}^{I}y+R_{13}^{I}z^{*}+R_{14}^{I}$$

(7b) $$y\prime =R_{21}^{I}x+R_{22}^{I}y+R_{23}^{I}z^{*}+R_{24}^{I}$$

(7c) $$s=R_{31}^{I}x+R_{32}^{I}y+R_{33}^{I}z^{*}+R_{34}^{I}$$

where we use R^*I*^_*ij*_ to denote the *ij*th element of $R^{-1}$. Eq. (7c) is easily solved for

(8) $$z^{*}=\frac{s-R_{31}^{I}x-R_{32}^{I}y-R_{34}^{I}}{R_{33}^{I}}$$

which can then be substituted in to Eqs. (7a), (7b).

The next step is to interpolate these values onto the regular *z*-coordinates of $S_{m}$, which we do separately for each column. A column is defined by a set of intensity values, g, and a set of $z^{*}$ coordinates. The interpolation is performed by fitting a set of cubic splines, one for each regular grid point that we want interpolated values for, to the observed values. First a “spline design matrix” $W^{\left(i\right)}$ (*i* for “irregular”) is created with as many rows as there are observations (i.e. the size of g and $z^{*}$) and as many columns as there are regular grid points. The elements of $W^{\left(i\right)}$ are given by $W_{\mathrm{ij}}^{\left(i\right)}=B(z_{i}^{*}-j)$ where

(9) $$\begin{array}{ccc} B(x)=\frac{2}{3}+\frac{x^{2}(|x|-2)}{2} & \mathrm{if} & |x|<1\\ B(x)=\frac{{\left(2-|x|\right)}^{3}}{6} & \mathrm{if} & 1\leq |x|<2\\ B(x)=0 & \mathrm{otherwise} & \end{array}$$

That means that there are some set of spline coefficients c such that

(10) $$g=W^{\left(i\right)}c$$

and that one can solve in a least squares sense for c by

(11) c^=(W(i)TW(i)+λD)−1W(i)Tg

where D is a regularisation matrix that minimises the integral of the second derivative of the interpolating function. From this one can obtain interpolated values for any arbitrary point *z* and in particular one can create a matrix $W^{\left(r\right)}$ (*r* for “regular”) with elements $W_{\mathrm{ij}}^{\left(r\right)}=B(i-j)$. Then

**Fig. 4:**
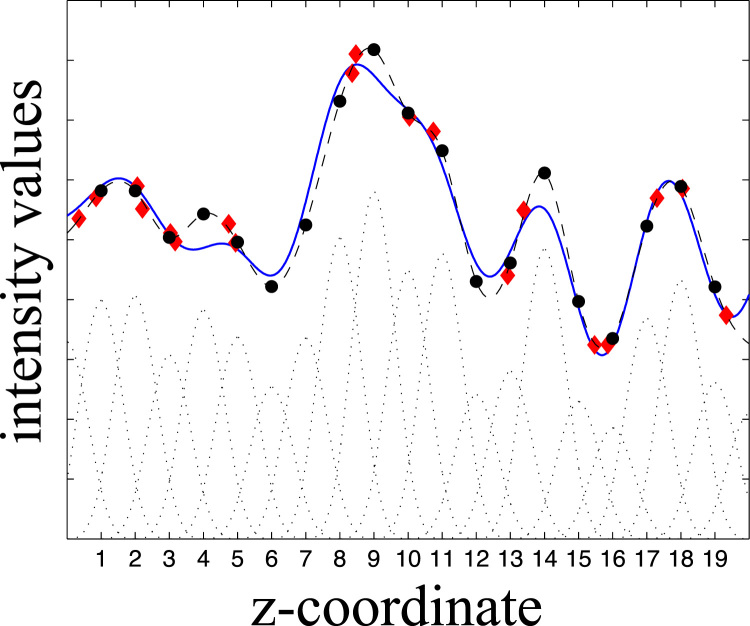
The figure shows a “true” function as a solid blue line. This function has been sampled at irregular intervals, and each sample (red diamond) also has some error (noise). A set of regularly spaced splines has been fitted to the red points and the black dotted lines at the bottom of the graph shows the spline functions multiplied with the pertinent coefficients c^. The black dashed line shows the resulting interpolating function obtained from summing all the splines for each value of *z*. The black circles represent the interpolated values for the interger *z* 1–19.

(12) y^=W(r)c^

are the interpolated values on the regular grid. The interpolation is summarised in Fig. 4.

#### 2.4.1 Adding predictions

The scheme described above will produce an interpolated volume regardless of the specifics of the movement parameters B. But when there are missing data, for the reasons described in Fig. 2, it can only fill those values in by finding the smoothest interpolating function, i.e. that which minimises the second derivative, across the gap. In previous work Andersson et al. (2016) we have suggested to replace missing data by the predictions from the Gaussian Process. In that case data was missing due to signal loss caused by subject movement, and here we extend the idea for data missing due to intra-volume movement. It is implemented by amending Eq. (11) to become a weighted least squares estimation with the weights calculated as given below.

**Fig. 5:**
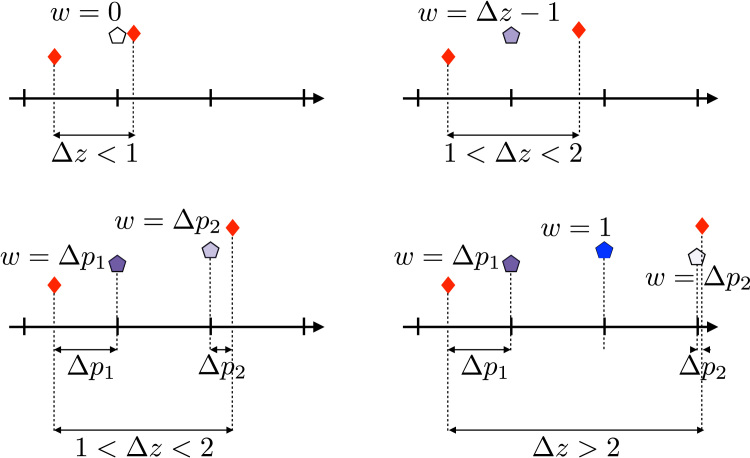
This figure explains how the weights are calculated when using the predictions in the irregular *z*-resampling. For all panels the red diamonds are observed points, the tick-marks are the regular grid-points for which one wants interpolated values and the pentagons are predicted values (which exist for all points on the regular grid). The weight given to any prediction is completely given by its bracketing observations. All distances in the figure are given in points on the regular grid. In the upper left corner is the case where the distance between the bracketing points (Δz) is less than one grid-point, in which case the prediction is not used at all. In the upper right corner is the case where 1<Δz<2 and where the bracketing points bracket a single grid-point. In this case the prediction will be given the weight Δz−1. The lower left corner still shows the case where 1<Δz<2, but now the points bracket two grid-points. In this case the weights for each prediction are given by the distance to the nearest bracketing observation. Note that this still means that the sum of weights for the two points is Δz−1. The bottom right panel shows the case where 2>Δz<3 and where the bracketing points bracket three grid-points. The middle prediction is now given the weight 1 and the weights for the other two points are still given by the distance to their nearest bracketing point.

The aim of the following heuristic is to ensure that whenever there are observations, they always take precedence over the predictions. The predictions are used solely to fill in the gaps that would otherwise force us to rely on λD. The predictions exist for all regular grid-points. That means that for each vector pair g and $z^{*}$ with size $N_{z}\times 1$ there are *N*_*s*_ predictions p that one potentially wants to use. To determine the weight *w*_*i*_ for each prediction *p*_*i*_ we use

(13) $$\begin{array}{ccccc} w_{i}=0 & \mathrm{if} & \Delta z<1 & & \\ w_{i}=\Delta z-1 & \mathrm{if} & 1<\Delta z<2 & \mathrm{and} & m=1\\ w_{i}=\Delta p_{i} & \mathrm{if} & \Delta z>1 & \mathrm{and} & m=2\\ w_{i}=\min(\Delta p_{i},1) & \mathrm{if} & \Delta z>2 & \mathrm{and} & m>2 \end{array}$$

where Δz is the distance between the observations bracketing *p*_*i*_, where $\Delta p_{i}$ is the distance between *p*_*i*_ and its closest neighbouring point and where *m* is the number of predictions between the two observations that bracket *p*_*i*_. Note that this heuristic means that the total weight to all predictions that fall between two bracketing points will always be min(0,Δz−1). This method for determining that weights is illustrated in Fig. 5.

With the weight vector w and the prediction vector p, Eq. (10) changes to

(14) $$\left[\begin{array}{c} g\\ \mathrm{diag}\{w\}p \end{array}\right]=\left[\begin{array}{c} W^{\left(i\right)}\\ \mathrm{diag}\{w\}W^{\left(r\right)} \end{array}\right]c$$

which is solved for interpolated values on the regular grid by

(15) y^=W(r)([W(i)Tdiag{w}W(r)T][W(i)diag{w}W(r)])−1[W(i)Tdiag{w}W(r)T][gdiag{w}p]

The important difference between Eqs. (12) and (15) is that in areas with no observations the former will yield the “smoothest” possible curve. In contrast, the latter will yield a curve that tends towards the predictions. In areas with sufficient observations the two versions will be very similar as the weights in w will be zero, or close to zero.

#### 2.4.2 Speed-up for the estimation of slice-to-volume movement

The resampling described above is high fidelity in that the interpolation is performed using splines, both for the initial in-plane interpolation and the subsequent cross-plane irregular interpolation. However, it is also computationally heavy because of the need to solve a linear system of 2N_z_ equations (Eq. (14)) for each column along *z* (of which there are $N_{x}\times N_{y}$). For that reason this strategy is only used for the final resampling of the data. During the iterative estimation of parameters (for distortions, movements and outliers) we instead use linear interpolation between points on g with locations given by $z^{*}$. No predictions are used during the estimation. Our initial testing showed that this did not significantly change the estimates compared to using the more sophisticated interpolation approach.

### 2.5 The algorithm

A description of the full algorithm is.

The last loop in the algorithm, about setting the “shape reference”, warrants some additional explanation. In one iteration eddy will update the parameters for each volume to “nudge” that volume towards the model held by the prediction maker. The model will be at an average point in the distortion-movement-parameter space. To make this concrete, imagine a data set consisting of two volumes where there has been some movement between them. In that case both volumes will end up at a location halfway between the two original volumes. Correspondingly, if the data set consists of two volume of which one has been affected by intra-volume movement, the end result will be two volumes with half of that intra-volume movement.

The “identification” step in the algorithm serves to find the volume that is the least affected by intra-volume motion, based on the assumption that if it is a good match for the prediction based on all volumes it should not have any intra-volume movement. To set this as a “shape reference” means to regress out all the non-constant movement terms estimated for that volume from all the other volumes.

## 3 Material and methods

### 3.1 Implementation

The method described in the present paper has been implemented in C++ as part of the eddy tool (Andersson and Sotiropoulos (2016) and http://fsl.fmrib.ox.ac.uk/fsl/fslwiki/EDDY) in FSL (see Smith et al. (2004) and http://fsl.fmrib.ox.ac.uk/fsl/fslwiki/). Some of the steps of the algorithm, in particular the calculation of the derivatives and the spline-interpolation on an irregular grid, are very computationally intense. They have therefore been parallelised for NVidia GPUs using CUDA (Farber, 2011). All the results in the present paper have been generated using the GPU version of the software.

### 3.2 Aim of analysis

When considering if intra-volume realignment is a good idea or not there are two main questions that need to be answered

• Can we estimate movement parameters reliably at a temporal resolution that is greater then “once per volume”?
• If so, will that lead to more accurate diffusion derived measures (e.g. FA) given that a different resampling strategy needs to be used?

The experiments below, and the analysis of them, are designed to attempt to answer both of those questions.

### 3.3 Simulations

#### 3.3.1 Data

We used the POSSUM MRI-simulator (Drobnjak et al., 2006, Drobnjak et al., 2010), extended to simulate diffusion weighted images (Graham et al., 2016). It should be noted that POSSUM is a highly realistic MRI simulator that simulates data in *k*-space by solving Bloch's and Maxwell's equations. This ensures that the images and their artefacts capture the key features of their real-world counterparts.

Data was simulated with known eddy currents, subject movement and signal dropout. The simulations were divided into single-band and multi-band (see for example (Moeller et al., 2010) or (Setsompop et al., 2012)) acquisitions with an MB-factor of three. For the MB acquisitions the assumption was that all slices within an MB-group were acquired with the subject in the exact same location. For all simulations there was a “ground truth” dataset that contained no noise, movement or outliers. A simulated dataset consisted of 12, interspersed, *b*=0 volumes, 32 *b*=700 volumes and 64 *b*=2000 volumes. The matrix size was 72×86×55 for the single-band acquisitions and 72×86×57 for the MB acquisitions. The voxel size was 2.5×2.5×2.5mm for both acquisitions. An interleaved slice/group ordering was used for both data sets. The directions for each non-zero shell were optimised on the half-sphere using Coulomb forces (Jones et al., 1999), and then randomly sign-swapped to achieve a “reasonable” distribution over the whole sphere. All simulations had eddy current-distortions commensurate with a Stejskal-Tanner diffusion weighting and no in-plane acceleration. Two different levels of subject movement were used. Movement parameters taken from an fMRI time-series of a healthy cooperative subject were obtained by registration of all volumes to the first using FLIRT (Jenkinson et al., 2002). These were interpolated onto 0.12 second intervals using cubic B-splines. This ensured that the movement that was “injected” into the simulated data was realistic. In addition to this we also simulated data for the case where the recorded movement was multiplied by three so as to create a data set with “bad” movement, but where its temporal evolution was still realistic. For the MB3 simulations the duration of the “acquisition” was only approximately one third of that of the single-band data, so the first third of the movement trace was used for simulating those data. The movement that was used is shown in figure S1 in the supplementary material. In the simulations no movement was injected into the first two volumes in order to ensure the existence of a “shape reference” (see Section 2.5) for the *b*=0 and the dwi volumes. Two SNR levels were used and were designed to yield an SNR of 20 and 40 for the *b*=0 volumes, corresponding to “poor” and “normal/good” SNR.

Data was generated both with and without movement-induced signal dropout (Storey and Frigo, 2007, Andersson et al., 2016). The outliers were generated by multiplying selected slices by a “dropout factor” randomly drawn between 0.1 and 0.9, where zero corresponds to complete dropout and 1.0 to no dropout. The total number of dropout slices was chosen so that approximately 3% of all slices were affected. That number was chosen to be commensurate with what is “typically” seen in datasets with “slightly difficult” subjects such as in the Whitehall Imaging substudy (Filippini et al., 2014) or in the study by Krogsrud et al. (2016). Since the outliers are caused by movement, the probability of a slice/MB-group being an outlier was set to be proportional to the temporal derivative of the true subject movement.

When reviewing the multi-band simulations we observed subtle bands of signal modulation in some slices of the volumes with the greatest amount of intra-volume movement. Our interpretation is that these are spin-history effects caused by the reduced time interval between successive excitations of the same spins. This was not observed in the single-band data where the repetition time was three times greater. We then repeated the multi-band simulations with a pause inserted between MB groups such that the repetition time was the same as for the single-band case, and these effects disappeared. Therefore we simulated data for both cases: With spin-history effects to see how our slice-to-volume method deals with that, and without spin-history effects (with increased repetition time) to be able to assess the intra-volume movement effects in isolation.

In total the simulations consisted of three types of acquisition, single-band, multi-band with short TR and multi-band with long TR. Each acquisition-type was simulated with “normal” and “large” movement. For each movement-type data was simulated with and without outliers and for each of those data was simulated with a b0-SNR of 20 and 40. Finally each simulation was repeated 10 times with different noise realisations. In total this produced 240 data sets of 108 volumes each.

#### 3.3.2 Analysis

Each simulated data set was corrected for eddy current-induced distortions, subject movement (Andersson and Sotiropoulos, 2016) and outliers with outlier-type set to “both” as described in Andersson et al. (2016). The movement correction was either volume-to-volume or slice-to-volume with 2, 4, 8 or 16 degrees of freedom per volume and movement parameter. For the slice-to-volume movement corrections, the movement-over-time regularisation *λ* (see Section 2.2.1) was set to 1 or 10. This yields a total of 9 corrections for each simulated data set and a sum total of 2160 runs.

We compared the known and estimated movement for each timepoint and calculated a per-volume-error as explained in Fig. 8. This was averaged for each movement parameter, separately for the *b*=0, *b*=700 and *b*=2000 volumes.

The “true” fractional anisotropy was calculated for the single-band and multi-band acquisitions. These were slightly different to each other since the exact location of the object was slightly different for 55 and for 57 slices. The calculations were performed using weighted least-squares fitting of log-transformed data (Basser et al., 1994). A “conservative” mask was created by performing brain extraction on the noise-free *b*=0 volume (Smith, 2002) followed by a one-voxel erosion to ensure that only intracerebral voxels were considered. The correlation between “true” and estimated FA was calculated across all intracerebral voxels for all corrected data sets.

### 3.4 Human data

#### 3.4.1 Data with deliberate movement

A healthy and experienced volunteer was scanned twice, in the same session, with an identical 60 direction (+ 5 *b*=0) diffusion scheme. For the first scan the subject was instructed to remain as still as possible. For the second scan the instruction was to perform controlled movements at intervals of roughly 10 volumes. The speed of the movement was to correspond to someone changing position (for example due to discomfort) in a sudden, though not jerky, fashion. The pair of “still+moving” scans was repeated thrice, once with a single-band and twice with an MB3 protocol. Common details for the two protocols were: Data was acquired on a Siemens Magnetom Prisma system with a 32-channel receive head coil. Diffusion encoding was performed using monopolar Stejskal-Tanner gradients. A single shell with 60 unique directions and a *b*-value of 1500 was acquired along with 5 interspersed *b*=0 volumes. The resolution was 2 mm isotropic and 6/8 partial Fourier was used. Details specific to the single-band scan were: 106×106 matrix size with 64 slices, posterior → anterior phase-encoding, in-plane acceleration was a factor 2 GRAPPA and the repetition time (TR) was 8.9 seconds. For the multi-band case the parameters were: 112×112 matrix size, anterior → posterior phase-encoding, no in-plane acceleration, MB3, 66 or 63 slices (22 or 21 MB-groups) and a TR of 2.8 s. Each diffusion scan was preceeded by a single *b*=0 volume acquired with opposing PE-direction, but with all other parameters identical.

The reason we acquired two sets of MB data was to compare odd and even number of MB-groups. In the Minnesota implementation of the multi-band sequence (Moeller et al., 2010) the slice-ordering differs quite radically depending on if an odd or even number of MB-groups is used (Center for Magnetic Resonance Research, University of Minnesota, 2012). For an odd number of MB-groups two adjacent slices will always be excited half a repetition time apart. This is not guaranteed for an even number. For example for the combination of MB3 and 66 slices, adjacent slices will be excited TR/4.4 apart. This means that for a one-slice out-of-plane movement such that the same slice is excited twice, the effective recovery time for a slice can go from 2.8 to 0.64 seconds for the even number of MB-groups case. Our hypothesis was therefore that the data acquired with an odd number of groups would have significantly less spin-history effects than the even data.

##### 3.4.1.1 Analysis

A fieldmap was calculated for each diffusion data set using the preceeding *b*=0 scan with opposing PE direction and the first *b*=0 volume of the diffusion data set using the FSL topup tool (Andersson et al., 2003). All data sets were subsequently corrected for susceptibility, eddy currents, outliers and subject movement using the eddy tool (Andersson and Sotiropoulos (2016) and Andersson et al. (2016)). The latter correction was performed using the volumetric model described in (Andersson and Sotiropoulos, 2016) and also using the slice-to-volume model described in the present paper with 8, 16 and 32 degrees of freedom per volume for the single band data and with 4, 8 and 16 degrees of freedom for the MB3 data. The assumption was that the data acquired when the subject was instructed to lie still would have very little motion, and that the volumetric model would be sufficient to correct what little motion there was. Hence, these data can be used as a form of “ground truth”. We then perform a voxelwise correlation between the pairs (with the same diffusion gradient) of “ground truth” and “motion” data. This was performed with the “motion” data corrected using the volumetric model or using the slice-to-volume model with different degrees of freedom.

#### 3.4.2 Whitehall imaging data

These data are from community-dwelling older adults and have been described in great detail in Filippini et al. (2014). Images were acquired on a 3 T Siemens Magnetom Verio system with a 32-channel receive head coil. Diffusion encoding was performed using monopolar Stejskal-Tanner gradients. A single shell with 60 unique directions and a *b*-value of 1500 was acquired along with 5 interspersed *b*=0 volumes. The resolution was 2 mm isotropic and the matrix size was 96×96×64 voxels. Phase-encoding was in the anterior → posterior direction and a factor of 2 in-plane acceleration (GRAPPA) was used. An additional single *b*=0 volume with phase-encoding posterior → anterior was acquired to allow for estimation of the susceptibility-induced off-resonance field.

Data was used from eight subjects who were identified (by N.F.) as “having moved a lot”.

##### 3.4.2.1 Analysis

The data was visually inspected for signs of intra-volume movement before and after correction.

## 4 Results

### 4.1 Simulations

In this, the main section of the paper, we present the results from the single-band simulations and from the multi-band simulations where the TR was artificially inflated to equal that of the single-band data. We do that in order to isolate the effects of the volume-to-volume versus the slice-to-volume movement models. However, the results for the short TR data were very similar and did not change any conclusions.

**Fig. 6:**
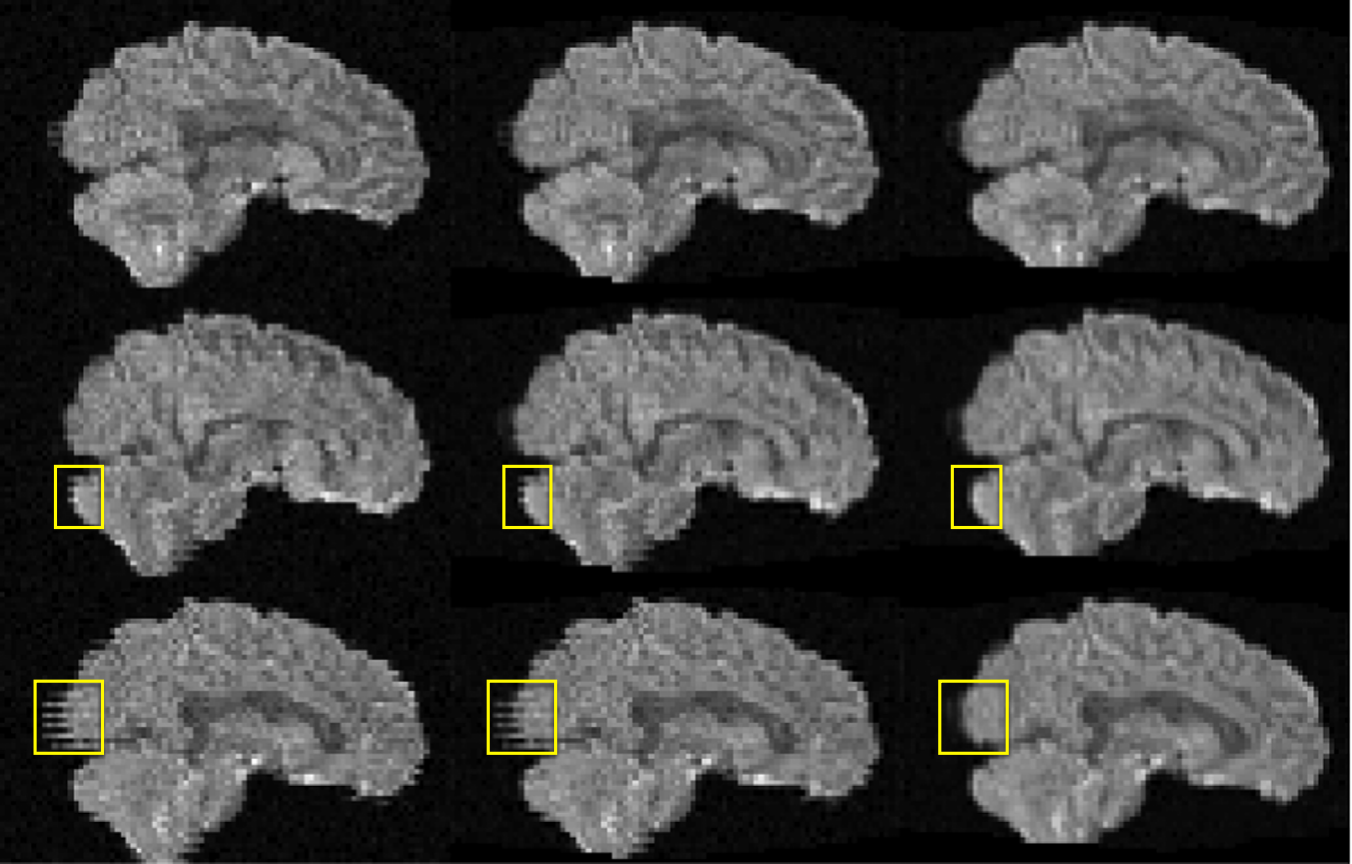
Examples of volumes with substantial (bottom row), some (middle row) and little (top row) intra-volume movement. They correspond to the last three volumes in the lower left panel of Fig. 7 (*b*=700). The leftmost column shows the raw images, the middle column after volumetric alignment and the rightmost column after slice-to-volume alignment. Yellow rectangles indicate places where the effects of intra-volume movement are particularly prominent.

Fig. 6 shows an example of simulated images with varying degrees of intra-volume movement. It can be seen that for the case with little intra-volume movement (top row) our slice-to-volume method yields results that are almost indistinguishable to those obtained with volumetric realignment. In contrast, for the case with substantial intra-volume movement (bottom row) the slice-to-volume method almost completely corrects for that. In the supplementary material there are movies showing simulated data before and after correction.

**Fig. 7:**
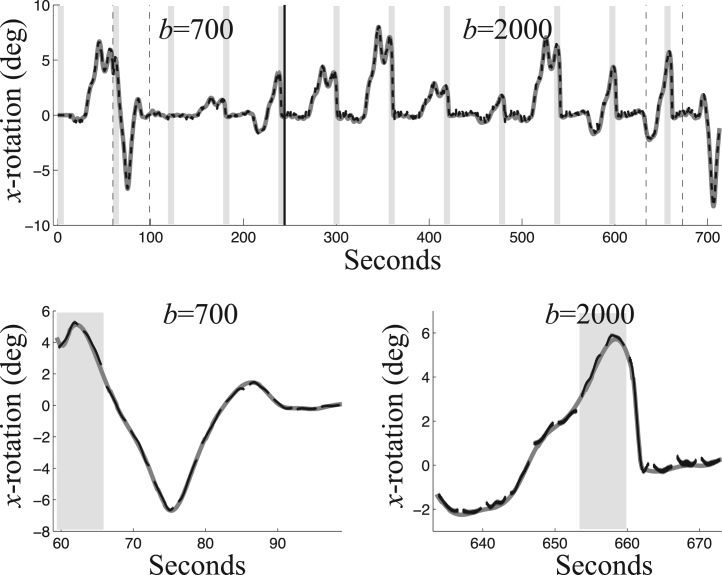
The top panel shows an example of the true (thick, solid, gray line) and estimated (dashed, black line) rotation around the *x*-axis as a function of time for the single-band, large motion, SNR=40 simulated data without dropout. These results were estimated using 16 DCT basis-functions and a regularisation *λ* of 1. The light gray vertical bands show the locations and extents of the *b*=0 volumes. The black vertical line shows the transition from *b*=700 to *b*=2000 among the DWIs. The lower panels shows two selected periods indicated by the vertical dashed lines in the top panel, one from the *b*=700 section and one from the *b*=2000 section. In the lower panels the estimated movement (in black) is only shown for the slices for which there is an appreciable amount (> 450 voxels) of brain present (slices 7–49 of 55). The estimates are the mean across all ten noise realisations of this simulation. The “thickness” of the black line is caused by this being an errorbar-plot where the errorbars are ± one standard deviation across the ten realisations.

**Fig. 8:**
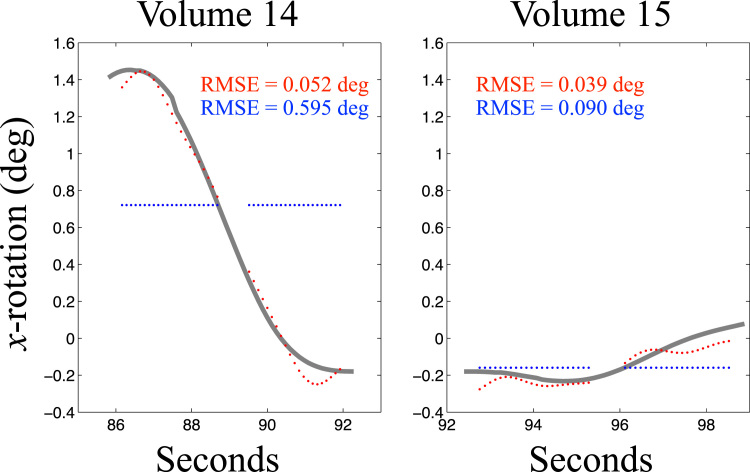
This figure demonstrates how the registration error was calculated. Shown in grey is the true rotation around the *x*-axis as a function of time for two volumes. These two volumes are the same as the two last volumes in the lower left panel of Fig. 7 and the top two rows in Fig. 6. They were chosen to demonstrate one volume with appreciable (left panel) and one with very little intra-volume movement (right panel). Each coloured dot shows the estimated rotation for one slice. Only slices with an appreciable amount of brain present were considered. The blue dots show the volumetric estimates and the red dots show the estimates using 16 DCT basis-functions and a regularisation *λ* of 1. For each dot the error is defined as the vertical distance to the truth. For a volume the RMSE is calculated as the square root of the mean of the squared vertical distances.

An example of estimated versus true movement over time is shown in Fig. 7 for rotation around the *x*-axis. The errors are similar for the other parameters, though on the whole it seems like the rotation around the *y*-axis is estimated with slightly lower precision than the other parameters. The estimates follow the true parameters quite well even in periods of rapid movement. Fig. 8 shows how registration error was evaluated.

**Fig. 9:**
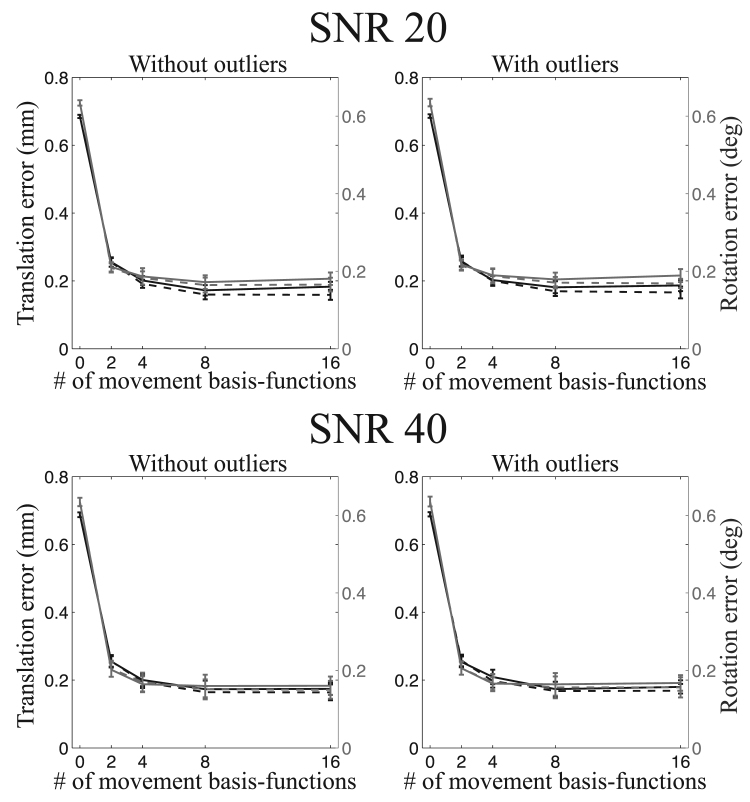
This figure shows the registration error for large movement and single-band acquisition. The translation errors (averaged over all axes) are shown in black and the rotation errors (also averaged around all axes) are shown in grey. The solid lines pertain to regularisation of the movement with λ=1 and the dashed lines with λ=10.

**Fig. 10:**
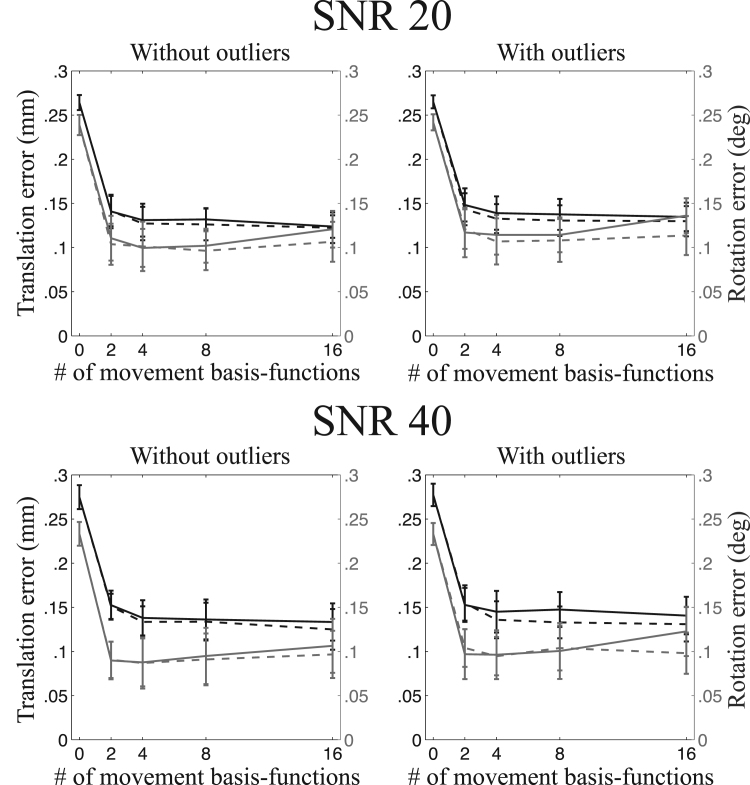
This figure shows the registration error for “normal” movement and single-band acquisition. The translation errors (averaged over all axes) are shown in black and the rotation errors (also averaged around all axes) are shown in grey. The solid lines pertain to regularisation of the movement with λ=1 and the dashed lines with λ=10.

**Fig. 11:**
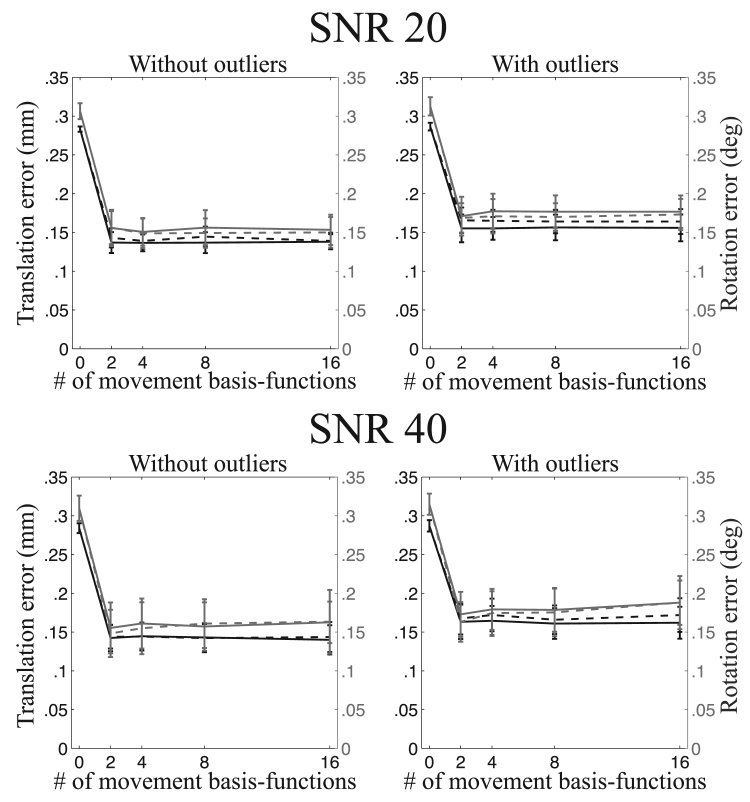
This figure shows the registration error for large movement and multi-band acquisition. The translation errors (averaged over all axes) are shown in black and the rotation errors (also averaged around all axes) are shown in grey. The solid lines pertain to regularisation of the movement with λ=1 and the dashed lines with λ=10.

**Fig. 12:**
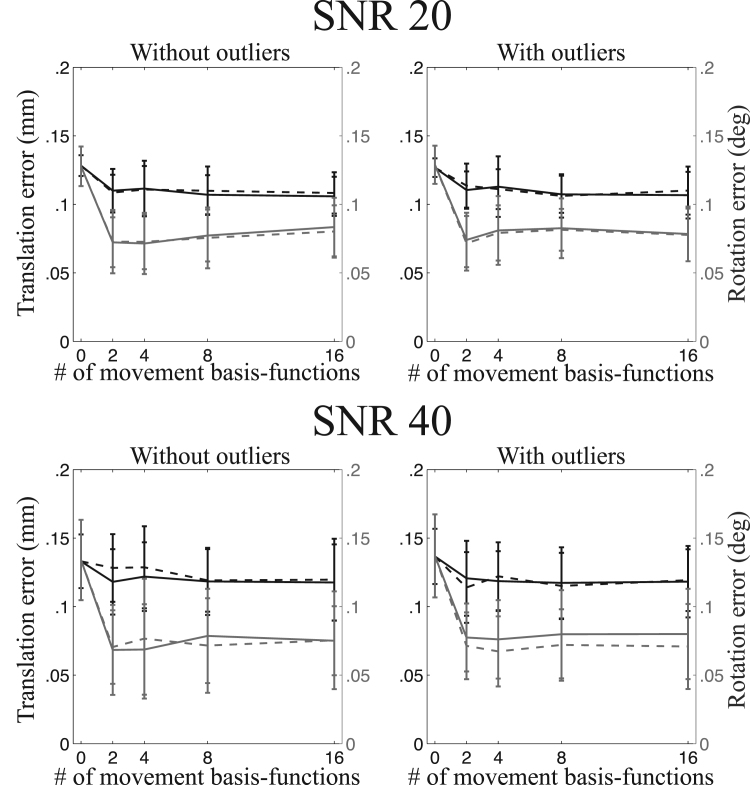
This figure shows the registration error for “normal” movement and multi-band acquisition. The translation errors (averaged over all axes) are shown in black and the rotation errors (also averaged around all axes) are shown in grey. The solid lines pertain to regularisation of the movement with λ=1 and the dashed lines with λ=10.

A summary of the registration errors collapsed over translation-directions, rotation-axes and image types are presented in Fig. 9, Fig. 10, Fig. 11, Fig. 12. It can be seen that going from a volumetric model to the slice-to-volume model reduces the registration errors to approximately 0.1–0.2 mm and 0.1–0.2 degrees for single-band data and to 0.07–0.15 mm and 0.07–0.15 degrees for multi-band data. It can also be seen that the volumetric model performs relatively better for multi-band compared to single-band data (compare for Fig. 9, Fig. 10, Fig. 11). This is caused by the improved ability to “freeze” any movement because of the shorter acquisition time for a single volume.

**Fig. 13:**
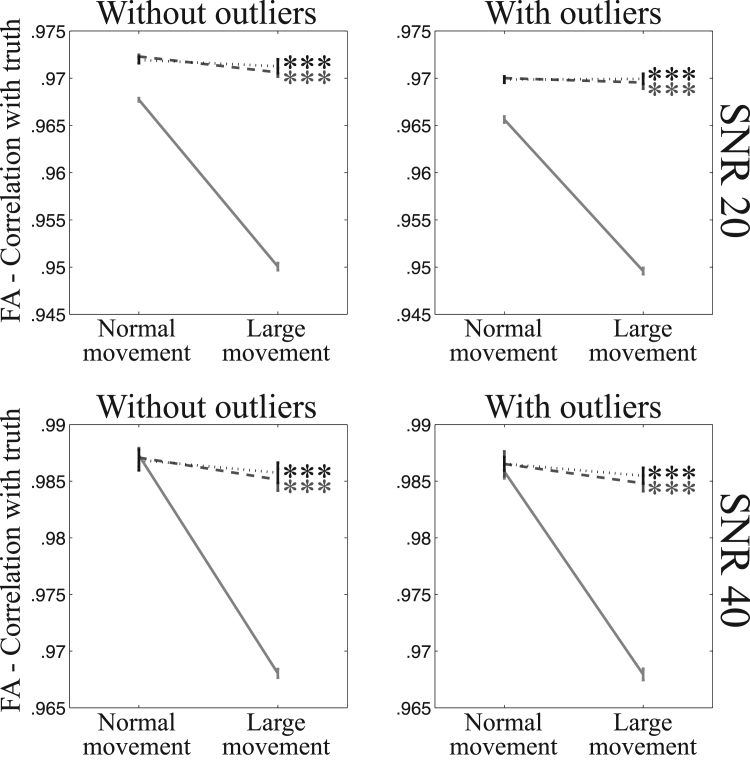
This figure shows the correlation between estimated and true FA for “normal” and “large” movements after correction of movements and distortions with eddy for the single-band data. The solid line shows results after correction using the volume-to-volume model and the dashed and dotted lines using the slice-to-volume model with 8 and 16 basis-functions respectively. A statistical test (testing for unequal slopes) was performed to assess whether the difference between the “normal” and “large” movements was greater for volume-to-volume correction compared to the pertinent slice-to-volume model. Significance was indicated with * (p≤0.05), ** (p≤0.01) or *** (p≤0.001).

**Fig. 14:**
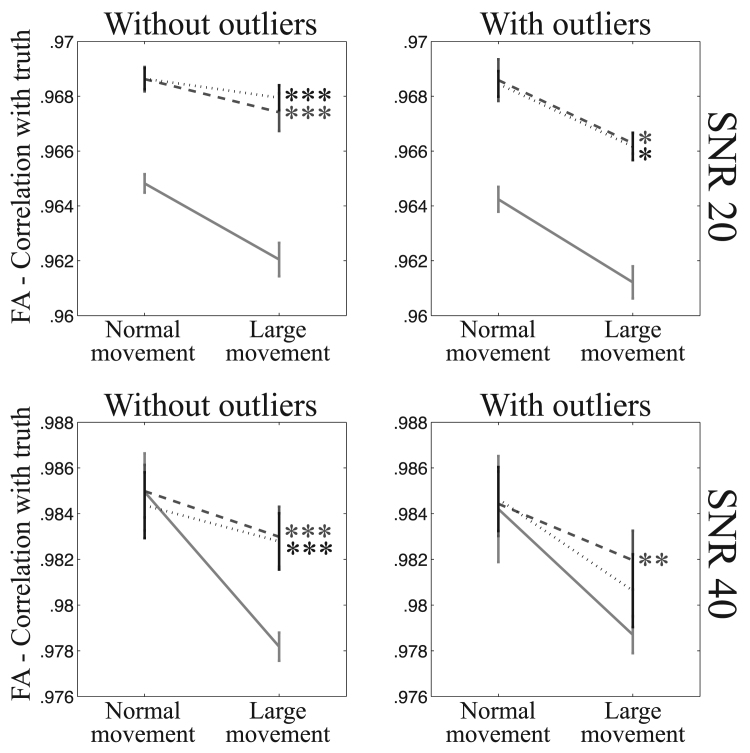
This figure shows the correlation between estimated and true FA for “normal” and “large” movements after correction of movements and distortions with eddy for the multi-band data. The solid line shows results after correction using the volume-to-volume model and the dashed and dotted lines using the slice-to-volume model with 8 and 16 basis-functions respectively. A statistical test (testing for unequal slopes) was performed to assess whether the difference between the “normal” and “large” movements was greater for volume-to-volume correction compared to the pertinent slice-to-volume model. Significance was indicated with * (p≤0.05), ** (p≤0.01) or *** (p≤0.001).

Fig. 13, Fig. 14 show the fidelity of FA calculations (as assessed through the correlation between true and estimated FA) for data simulated with “normal” and “large” movement. It is clear that for single-band data there is a large difference in fidelity that is almost completely resolved by the intra-volume model. The advantage is less clear for multi-band data, but that is mainly due to the difference (between “normal” and “large” movement) being much smaller to begin with.

An even more detailed view of the simulation results are offered by tables S1 to S16 in the supplementary material. Additionally we show the results for the short TR data in figures S2 to S5 in the supplementary material.

### 4.2 Human data

#### 4.2.1 Data with deliberate movement

**Fig. 15:**
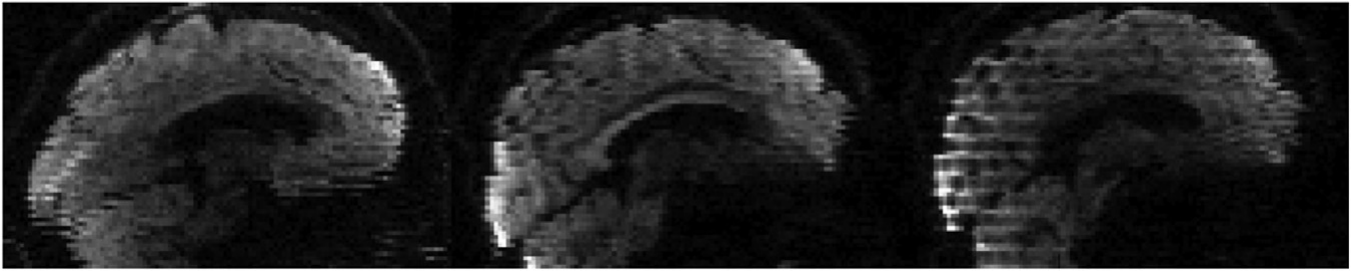
Examples, from the data with deliberate movement, of volumes corrupted by intra-volume movement (mainly *x*-rotation). From left to right single band, MB3 with odd number of groups and MB3 with even number of groups.

An inspection of the movement parameters for the “still” data sets confirmed that the subject had remained very still during these acquisitions. Both the volume-to-volume and the slice/group-to-slice/group movement was very small, so it was concluded that the volumetric model was fully sufficient to correct for movement. The movement parameters for the “motion” data on the other hand showed large movements between as well as within volumes. This movement was mainly rotations around the *x* and *z*-axes, as had been the intention. Examples of volumes affected by intra-volume movement are shown in Fig. 15. In each case they represent the dwi volume with the greatest intra-volume rotation around the *x*-axis. In all three cases one can see the telltale jagged edges at the back and front of the brain. In addition one can see a clear intensity modulation caused by spin-history effects for the MB data with an even number of groups.

There are also before and after movies of these data in the supplementary material, and we recommend taking a look at these.

**Fig. 16:**
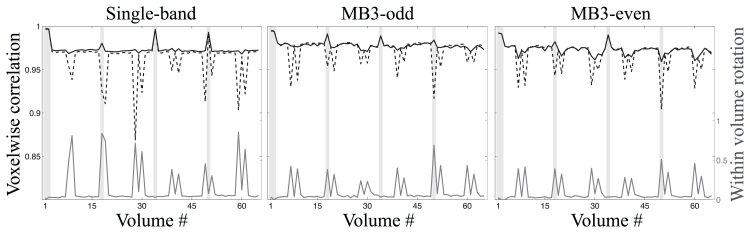
For each panel the correlation between ground truth and the “motion” data is shown as a dashed black line for the volumetric correction model and as a solid black line for the slice-to-volume correction model. Also shown, as a solid dark grey line, is a statistic for the amount of intra-volume movement in each volume. The *y*-axis on the left pertains to the correlation between paired volumes and the *y*-axis on the right to the intra-volume movement. The locations of the *b*=0 volumes are indicated by light grey vertical bands.

For the comparison of the “motion” data to ground truth (as represented by the “still” data) we calculated the correlation between corresponding pairs from the two data sets. In addition, the amount of intra-volume movement was assessed as the within volume standard deviation of the individual rotation parameters, average across the three rotations. This was used as a summary statistic for intra-volume movement. The results of this can be seen in Fig. 16. From the results in Fig. 16 we also estimated how well the slice-to-volume model did in reversing the effects of intra-volume movement. We did that by calculating the average correlation (separately for *b*=0 and dwi volumes) for the volumes not affected by intravolume movement (rotation standard deviation <0.1 degrees) and compared that to the average correlation, using a volumetric model, for the affected volumes. We then calculated how large a percentage of that difference was reversed by using a slice-to-volume model. The reduction was 102%, 85% and 68% for single band, MB-odd and MB-even respectively. A 102% is admittedly “too good”, but we interpret that as the correction being close to 100%. Furthermore, we do believe the drop from 85% to 68% when going from odd to even number of MB-groups is a real effect. It is very much in accordance with the observed, strong, spin-history effects for the even case, an effect that we currently have no means of correcting.

We also calculated the correlation between the FA estimated from the “still” data and from the “motion” data corrected with either the volumetric or the slice-to-volume model. For all three sessions the correlation increased when using the slice-to-volume correction and the numbers were 0.965→0.980, 0.974→0.982 and 0.969→0.979 for single-band, MB3-odd and MB3-even respectively. Comparing this to Fig. 13, Fig. 14 one sees that these increases are in good agreement with the results from the simulations.

All the results we present here are for 16 degrees of freedom for the single-band data and with 8 degrees of freedom for the MB3 data. The results for 8 and 32 degrees of freedom for single-band and 4 and 16 degrees of freedom for MB3 data are very similar so we chose not to show them here.

Several things are immediately clear from Fig. 15, Fig. 16: (i) The correlation to ground truth is strongly impaired by intra-volume movement when using a volumetric correction model, (ii) the adverse effects are less for multi-band data, presumably for its greater ability to “freeze” movement in time, (iii) the exact slice ordering of multi-band sequences has a major impact on how motion causes spin-history effects and (iv) our slice-to-volume correction is very successful in counteracting the adverse effects of intra-volume movement, but less so in the presence of spin-history effects.

#### 4.2.2 Whitehall imaging data

All the human data confirmed the conclusions from the simulations. For all subjects with signs of intra-volume movement the slice-to-volume movement model improved the visual appearance of data after correction compared to the volume-to-volume model.

**Fig. 17:**
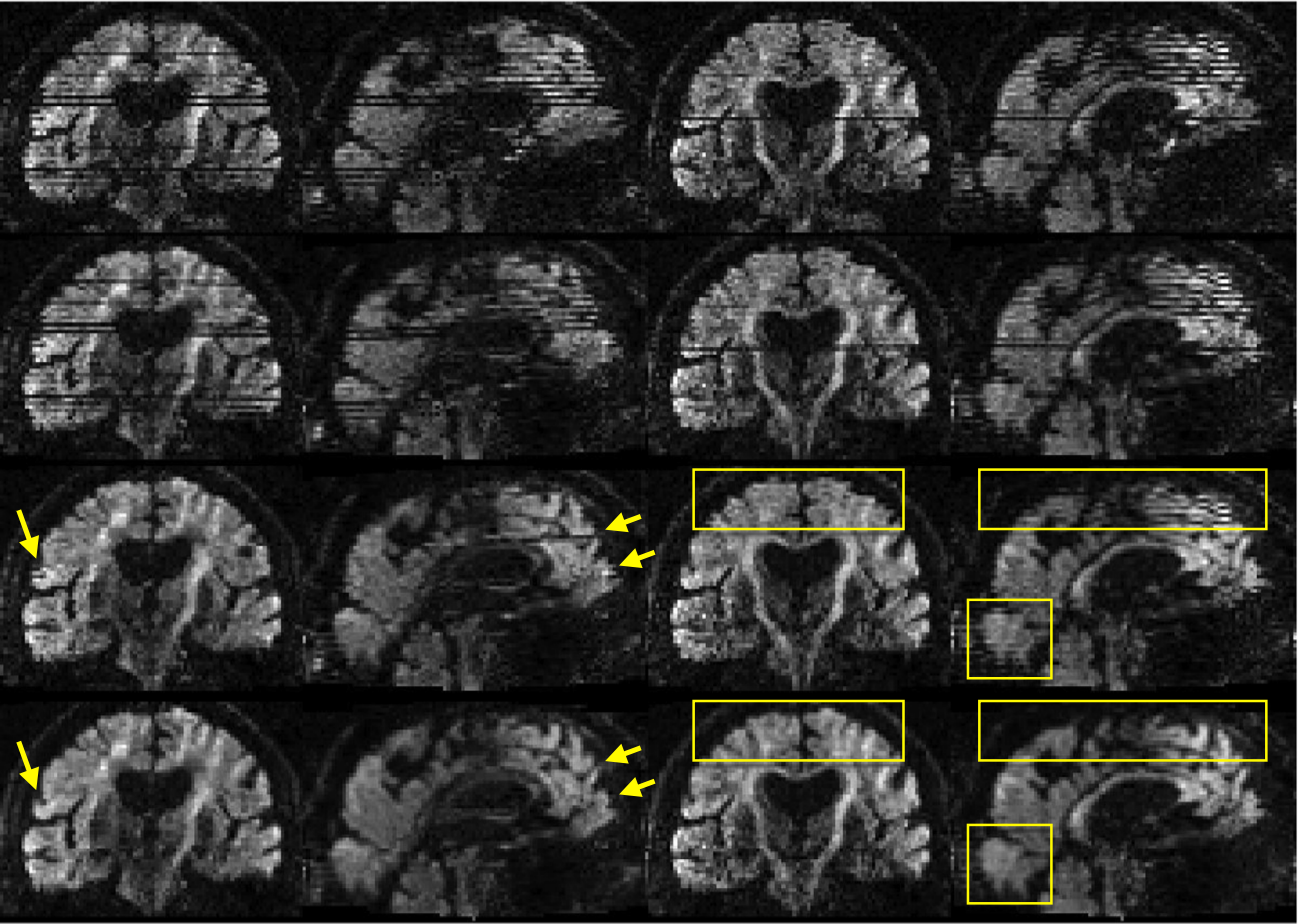
The two leftmost columns show one volume, and the two rightmost columns another volume, from an elderly Whitehall subject that moved a lot. The volume on the left was chosen to demonstrate the case where both intra-volume movement effects and movement-induced signal dropout are present. The volume on the right represents a volume with predominantely intra-volume movement effects. The top row shows the original data. The second row shows data after correction for susceptibility, eddy currents and volume-to-volume movement. For the third row data was additionally corrected for outliers. In the final row data was corrected for all of the above, and additionally using the slice-to-volume model with 16 degrees of freedom and a movement regularisation *λ* of 1. Yellow arrows and rectangles are used to highlight areas of particular interest.

An example of images before and after correction are shown in Fig. 17. However, for assessing these kinds of corrections a movie is much more illustrative and we strongly encourage readers to view the corresponding movie in the supplementary material.

## 5 Discussion

### 5.1 Registration error

The experiments assessing the accuracy of the estimated movement parameters show that the intra-volume model performed better than the volume-to-volume model for all cases. It also shows that the gains are greater for larger movements and greater for single-band than multi-band data. Both these results are completely expected as larger movement also means larger intra-volume movement and multi-band means that there is less time for movement to evolve over the duration of a volume acquisition. It is interesting to compare Fig. 10, Fig. 11 to Fig. 9, noticing that reducing the movement by a factor of three and reducing the time it takes to acquire a volume by a factor of three has approximately the same impact on the accuracy.

### 5.2 Impact on group-wise comparisons of scalar diffusion parameters

Probably the most important result of the paper is that shown in Fig. 13. It demonstrates that for single-band data there is a substantial difference in the veracity of FA estimated from subjects with a “normal” amount of movement compared to subjects that move “a lot” when data was pre-processed with a volumetric movement model. This observation goes a long way towards explaining findings such as those in Yendiki et al. (2014). They found that by stratifying a group of normal children by how much they moved in the scanner they saw significant differences in FA between the low- and high-movement groups. Fig. 13 further shows that when pre-processing data with a slice-to-volume model the difference in accuracy is reduced very substantially.

The difference between the two movement models is much smaller for multi-band data, as can be seen in Fig. 14. However, this does not mean that the slice-to-volume model has worse performance for multi-band data. On the contrary, Fig. 9, Fig. 10, Fig. 11, Fig. 12 indicate that the registration accuracy is greater for multi-band data. The result can be explained by the improved ability of multi-band to freeze movement in time, resulting in a smaller difference between normal and large movements when using the volume-to-volume model. From that one can expect that with the increased use of multi-band acquisition there will be less problems with movement-induced false positives in group comparisons, even without using slice-to-volume pre-processing.

### 5.3 When should one do intra-volume registration?

Our validation has shown that the advantages of intra-volume registration are the greatest when subjects move a lot and/or when acquiring a single volume takes a relatively long time (i.e. for single-band data). Importantly it has also demonstrated that for the simulations and data we have used, it does no harm. We would therefore argue that by virtue of that, and by virtue of it being a more realistic model for how movement occurs, the slice-to-volume model should always be used. In particular we believe that it should be used when performing a group-comparison (using for example TBSS, Smith et al. (2006)).

### 5.4 How many degrees of freedom should be used?

Because of the inherent smoothness of the movement trace that was injected into our simulations, we cannot make a recommendation directly from those results. The results indicate that 2-4 basis-functions (over and above the constant) are sufficient to track the movement. But that result is unlikely to generalise to all cases as it will depend strongly on the specific movement in any given data set. The important result from our simulations is that for all cases (single-band or multi-band, normal or large movement) the registration accuracy remained significantly better than the volume-to-volume approach for up to 16 degrees of freedom. In fact it remained largely unchanged for up to 16 degrees of freedom for all cases except for the single-band data with normal movement. In that case there was a trend towards lower accuracy for 16 than for 8 degrees of freedom when a regularisation *λ* of 1 was used. This means that it is “safe” to use at least as high as 16 degrees of freedom for the movement.

The results from the human data with deliberate movement showed no appreciable differences between 8, 16 or 32 degrees of freedom for the single-band data and no appreciable differences between 4, 8 or 16 degrees of freedom for the MB3 data.

Our experience of actual data with problematic movement is that there is little or no apparent improvement if more than 16 degrees of freedom are used. Though it is also the case that we have not noted any deterioration of results even when going up to the same number of degrees of freedom as the number of slices/groups.

### 5.5 Spin-history effects for multi-band data

An incidental finding in this study was that while multi-band acquisition reduces the problem of intra-volume movement, it also suffers from spin-history effects in the presence of large subject movements. This was first noticed in our simulations, and that finding led us to acquire human data with deliberate movement with two different layouts (in time) of MB3 groups. The results from this (see Fig. 15) show clearly that the exact details of the timing of multi-band groups can have an impact.

It is not surprising that multi-band acquisition can lead to spin-history effects, given that it reduces the time between consecutive excitations by the MB factor. Clearly the ability of multi-band to better “freeze the movement” is a very good thing when scanning populations that are problematic from a movement perspective. But in order to achieve its full potential we believe that these issues need to be better explored, and that an optimal slice/group-ordering that minimises spin-history effects for typical movement traces need to be found.

### 5.6 Relationship to earlier work

The unique aspect of the work presented here is the insertion of a slice-to-volume movement model into a coherent framework where both susceptibility- and eddy current-induced distortions are modeled along with the movement. Previous work have either ignored distortions (Kim et al., 1999, Bannister et al., 2007) or modeled only susceptibility-induced distortions (Yeo et al., 2008), though the latter pertained to fMRI so only susceptibility-induced distortions were present.

Other related methods are those used in fetal imaging where scattered slices are mapped into a volume by means of an intermediary volumetric model (Jiang et al., 2007) or through intersection matching (Kim et al., 2010). However, these are also quite different in the sense that they are using oversampled data acquired with a single contrast. Furthermore they use an imaging sequence that is relatively insensitive to off-resonance effects, so they don't need to take distortions into account.

There are also methods for motion correction of diffusion images of the fetal brain (Jiang et al., 2009, Oubel et al., 2012, Fogtmann et al., 2014) with similarities to the suggested method. There are also important differences in that these either ignore off-resonance distortions completely (Jiang et al. (2009) and Fogtmann et al. (2014)) or consider only eddy current-induced distortions (Oubel et al., 2012). This may be sufficient for fetal imaging where there are no air-filled sinuses or ear canals that disrupt the field, and if an eddy current-nulled sequence is used to minimise eddy currents (Reese et al., 2003). However, in the general case all of these sources of artefacts need to be considered.

### 5.7 Conclusion

We have augmented our framework for simultaneous correction of susceptibility- and eddy current-induced distortions and subject movement effects with a slice-to-volume movement model. It yields very accurate estimates of movement over time, and it almost completely reverses the negative effects that intra-volume movement has on the ability to accurately estimate FA.

## Appendix A Terms and definitions

In this appendix we will define some of the notation used in the main paper. Furthermore, we extend the simplified description of the inverse spatial model in Section 2.4 to include off-resonance effects.

### A.1 Definitions

***m*: Reference/model space.**: Is used to denote a space given by a FOV and an orientation in which the images are free of any distortions or effects of movement.
***f*: Observed/Acquired image.**: Is used to denote the observed image, and the space associated with it, so that for example *f*_*i*_ denotes the *i*th acquisition/volume in the data set. Note that each observation is associated with a unique space given by the eddy currents and subject movement for that particular volume.
a: **Acquisition parameters.**: The acquisition parameters that are relevant for eddy are the direction of the phase encoding and the total readout time (here defined as the time between the acquisition of the centre of the first and last echoes). Different scans in a set of diffusion images may have different acquisition parameters so $a_{i}$ denotes the acquisition parameters for the *i*th scan.
β**and**e(β)**: Eddy current-induced field.**: β is a set of parameters that uniquely define an EC-induced field e(β). Each scan is associated with a unique set of EC parameters so $\beta_{i}$ denotes the EC parameters for the *i*th scan.
***h*: Susceptibility induced off resonance field (Hz).**: This is not calculated by eddy, but will be used if supplied as an input.
$\omega (\psi ,r_{i}(s))$**: Rigid body transformed off-resonance field (Hz).**: This is the off-resonance field *ψ* for slice *s* after rigid body transformation with the parameters $p_{i}$.
d(ψ,a)**: Voxel displacement field.**: Given a field (*ψ*) in Hz and the acquisition parameters (a=[pt]) the voxel displacement field is denoted by d(ψ,a).

### A.2 Inverse spatial model

In the main text the transform of a scan *f*_*i*_ into the regular grid of the model-space *m* is given by Eqs. (6), (7a), (7b), (7c), and ignores any distortions. In order to introduce the distortions we first calculate

(A1) $$z^{*}=\frac{s-R_{31}^{I}x-R_{32}^{I}y-R_{34}^{I}}{R_{33}^{I}}$$

and

(A2a) $$x\prime =R_{11}^{I}x+R_{12}^{I}y+R_{13}^{I}z^{*}+R_{14}^{I}$$

(A2b) $$y\prime =R_{21}^{I}x+R_{22}^{I}y+R_{23}^{I}z^{*}+R_{24}^{I}$$

for each slice *s*, where R^*I*^_*ij*_ denotes the *ij*th element of $R{\left(s\right)}^{-1}$.

Secondly a slice-wise disjoint displacement field is created by calculating

(A3) $$d(s)=d(h(\{x,y,z^{*}\}(s))+\omega (e(\beta_{i}),\{x\prime ,y\prime ,s\}(s)),a_{i})$$

for every slice *s* and stacking these into a volume d. The notation $h(\{x,y,z^{*}\}(s))$ means that the contribution from *h* for coordinate [xys] in d is obtained by sampling *h* at the coordinate $\left[x\;y\;z^{*}\right]$. Similarly, the contribution from *ω* is obtained by sampling it at [x′y′s]. Both *h* and $e(\beta_{i}))$ are defined in the model-space *m*, so will exist for all coordinates and can be re-sampled using regular spline interpolation.

A new set of x′- and y′-coordinates for the 2D resampling of *f*_*i*_ onto *m* are calculated using

(A4a) $$x\prime =R_{11}^{I}x+R_{12}^{I}y+R_{13}^{I}z^{*}+R_{14}^{I}+d_{x}(x,y,z)$$

(A4b) $$y\prime =R_{21}^{I}x+R_{22}^{I}y+R_{23}^{I}z^{*}+R_{24}^{I}+d_{y}(x,y,z)$$

The final 1D resampling onto the regular *z*-grid is performed using the $z^{*}$ coordinates (Eq. (A1)) as described in Section 2.4 in the main text. The intensity modulation is performed as described in Andersson and Sotiropoulos (2016) using the Jacobian calculated from d(s).

### A.3 Movies

Figs. 6 and 17 in the main text show before and after slice-to-volume registration for simulated and real data respectively. A better perception of how the registration has worked is offered by these movies. Below we show stills from five movies, one on simulated data, three on data acquired on a subject performing deliberate movement and one on data from a subject in the Whitehall cohort.

## Appendix A Supplementary data

Video 1 The movie shows two coronal and two sagittal slices from each volume of a simulated data set with SNR 40, large movement and no outliers. The top row shows the original data, the middle row after correction with the volume-to-volume model and the bottom row after correction with the slice-to-volume model.

Video 2 Each row shows two coronal and two sagittal slices through a single band diffusion scan. The top row shows raw data from the scan where the subject was asked to perform deliberate movements and the bottom row shows data from the scan where the subject was asked to remain as still as possible, corrected for susceptibility, eddy-currents and subject movement using a volumetric model. The middle row shows the scan with deliberate movement after correction for distortions and subject movement using the slice-to-volume model.

Video 3 Each row shows two coronal and two sagittal slices through an MB3-odd diffusion scan. The top row shows raw data from the scan where the subject was asked to perform deliberate movements and the bottom row shows data from the scan where the subject was asked to remain as still as possible, corrected for susceptibility, eddy-currents and subject movement using a volumetric model. The middle row shows the scan with deliberate movement after correction for distortions and subject movement using the slice-to-volume model.

Video 4 Each row shows two coronal and two sagittal slices through an MB3-even diffusion scan. The top row shows raw data from the scan where the subject was asked to perform deliberate movements and the bottom row shows data from the scan where the subject was asked to remain as still as possible, corrected for susceptibility, eddy-currents and subject movement using a volumetric model. The middle row shows the scan with deliberate movement after correction for distortions and subject movement using the slice-to-volume model.

Video 5 The movie shows to coronal and two sagittal slices from each volume of a data set acquired on an elderly subject as part of the Whitehall study. The top row shows the original data. The second row shows the data after correction with the volume-to-volume model. The third row shows data after correction with the volume-to-volume model *and* outlier rejection and replacement (Andersson et al., 2016). The fourth row shows data after correction with the slice-to-volume model *and* outlier rejection and replacement.

Application 1
