# Supplementary figures and images for: Towards a comprehensive framework for movement and distortion correction of diffusion MR images: Within volume movement
Source: Neuroimage. 2017 May 15;152:450–66. doi: 10.1016/j.neuroimage.2017.02.085 (PMC5445723; doi:10.1016/j.neuroimage.2017.02.085)

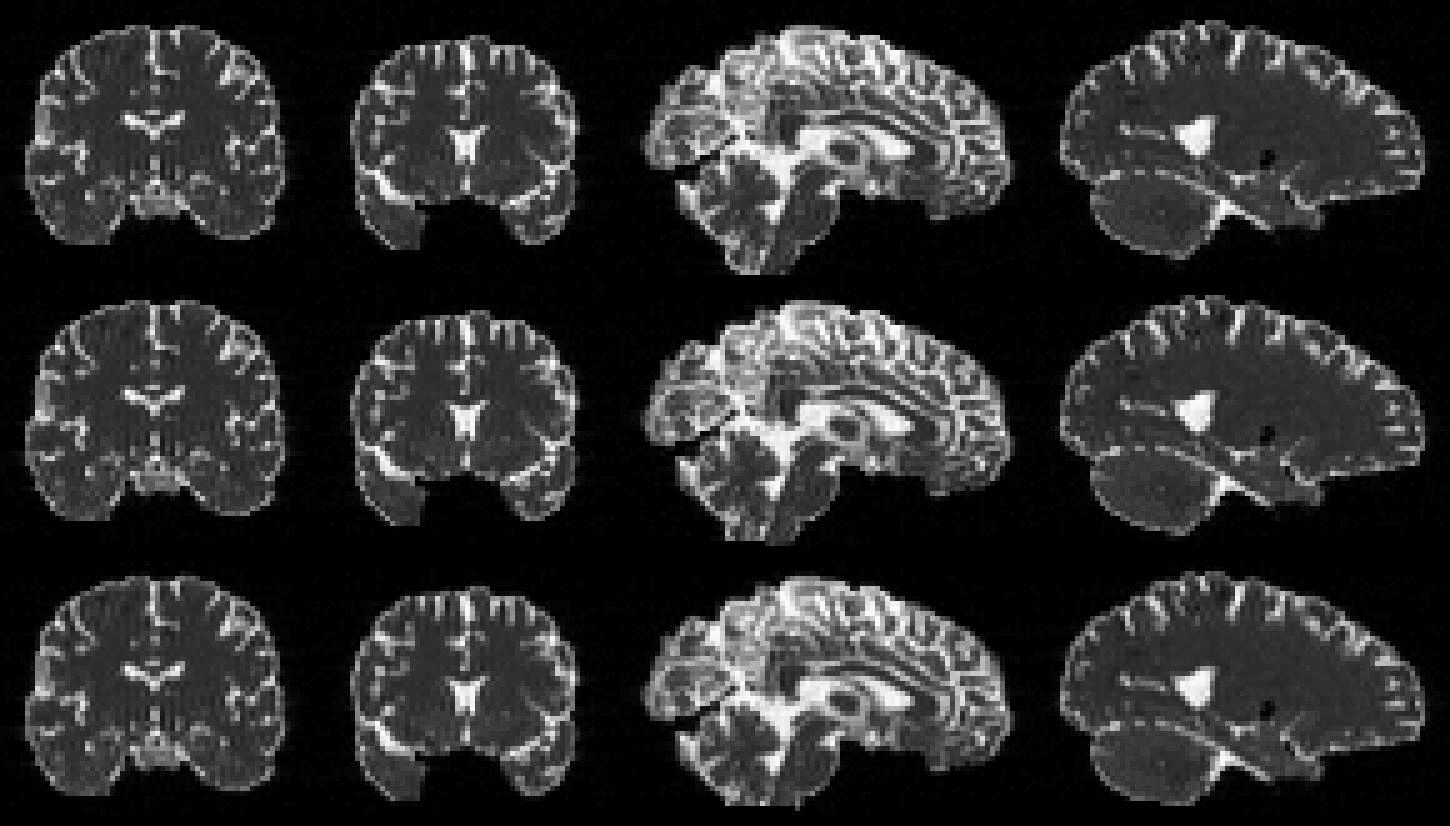

Supplement: Video 1 — The movie shows two coronal and two sagittal slices from each volume of a simulated data set with SNR 40, large movement and no outliers. The top row shows the original data, the middle row after correction with the volume-to-volume model and the bottom row after correction with the slice-to-volume model. [file mmc1.gif]

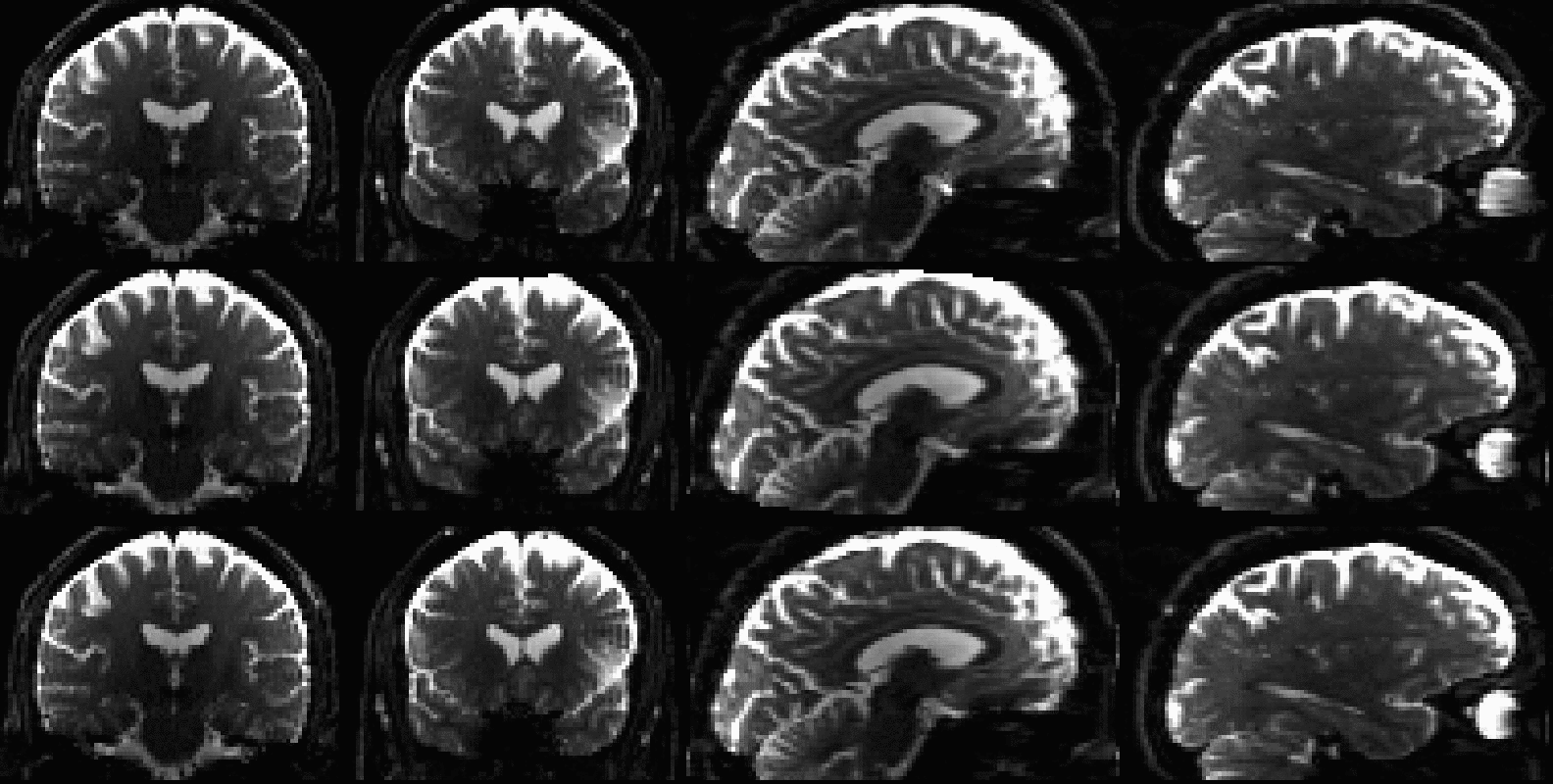

Supplement: Video 2 — Each row shows two coronal and two sagittal slices through a single band diffusion scan. The top row shows raw data from the scan where the subject was asked to perform deliberate movements and the bottom row shows data from the scan where the subject was asked to remain as still as possible, corrected for susceptibility, eddy-currents and subject movement using a volumetric model. The middle row shows the scan with deliberate movement after correction for distortions and subject movement using the slice-to-volume model. [file mmc2.gif]

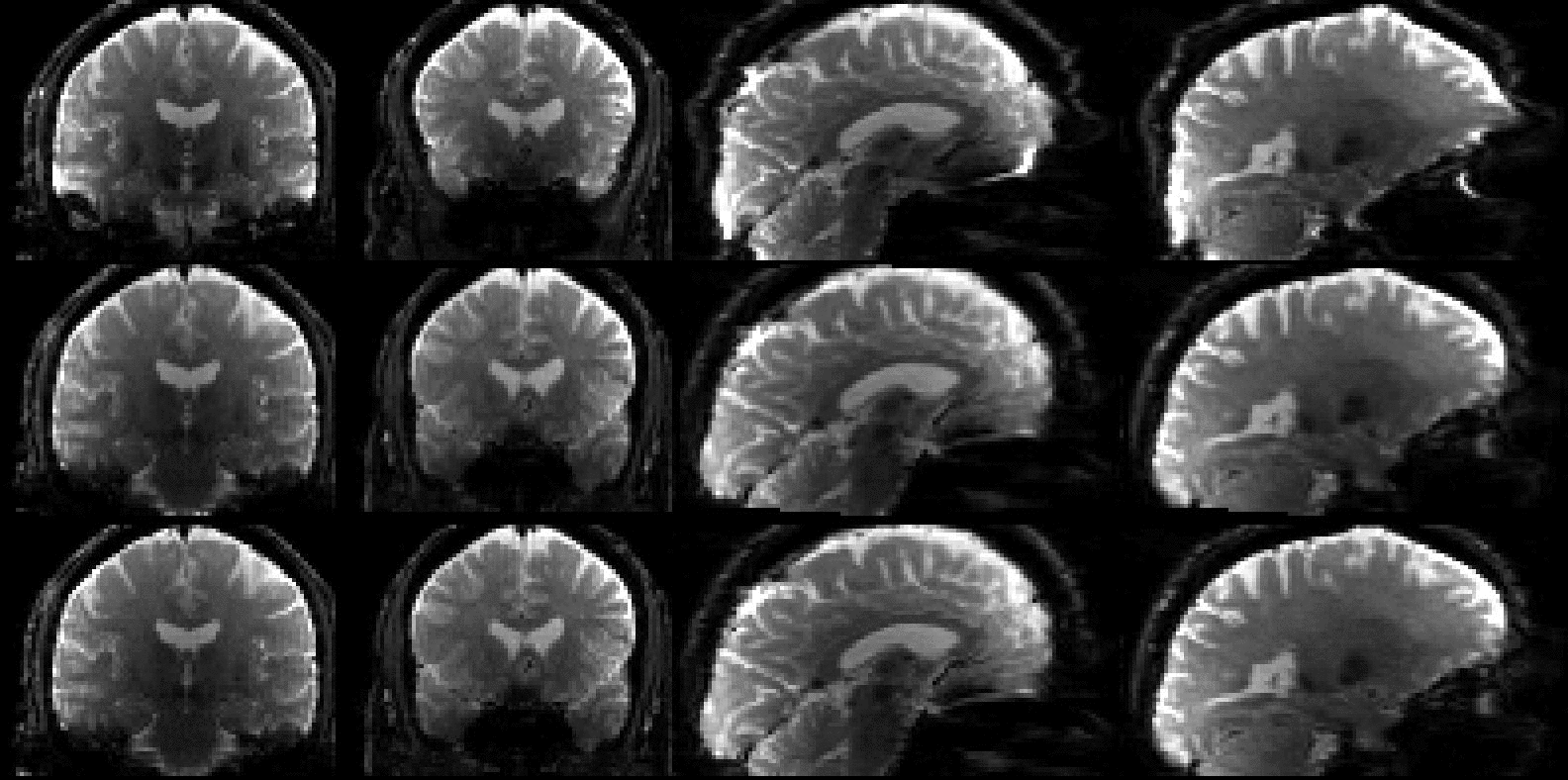

Supplement: Video 3 — Each row shows two coronal and two sagittal slices through an MB3-odd diffusion scan. The top row shows raw data from the scan where the subject was asked to perform deliberate movements and the bottom row shows data from the scan where the subject was asked to remain as still as possible, corrected for susceptibility, eddy-currents and subject movement using a volumetric model. The middle row shows the scan with deliberate movement after correction for distortions and subject movement using the slice-to-volume model. [file mmc3.gif]

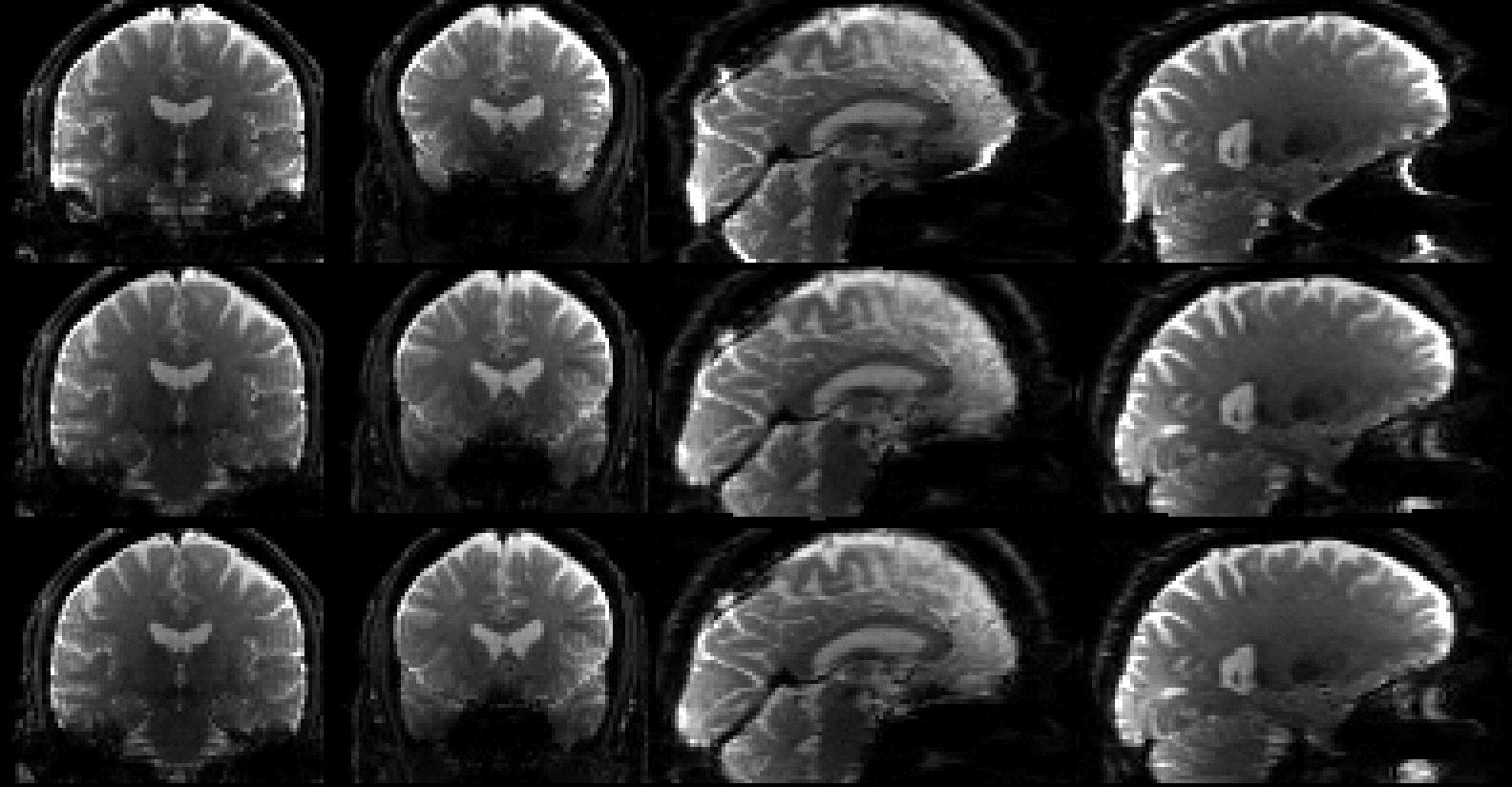

Supplement: Video 4 — Each row shows two coronal and two sagittal slices through an MB3-even diffusion scan. The top row shows raw data from the scan where the subject was asked to perform deliberate movements and the bottom row shows data from the scan where the subject was asked to remain as still as possible, corrected for susceptibility, eddy-currents and subject movement using a volumetric model. The middle row shows the scan with deliberate movement after correction for distortions and subject movement using the slice-to-volume model. [file mmc4.gif]

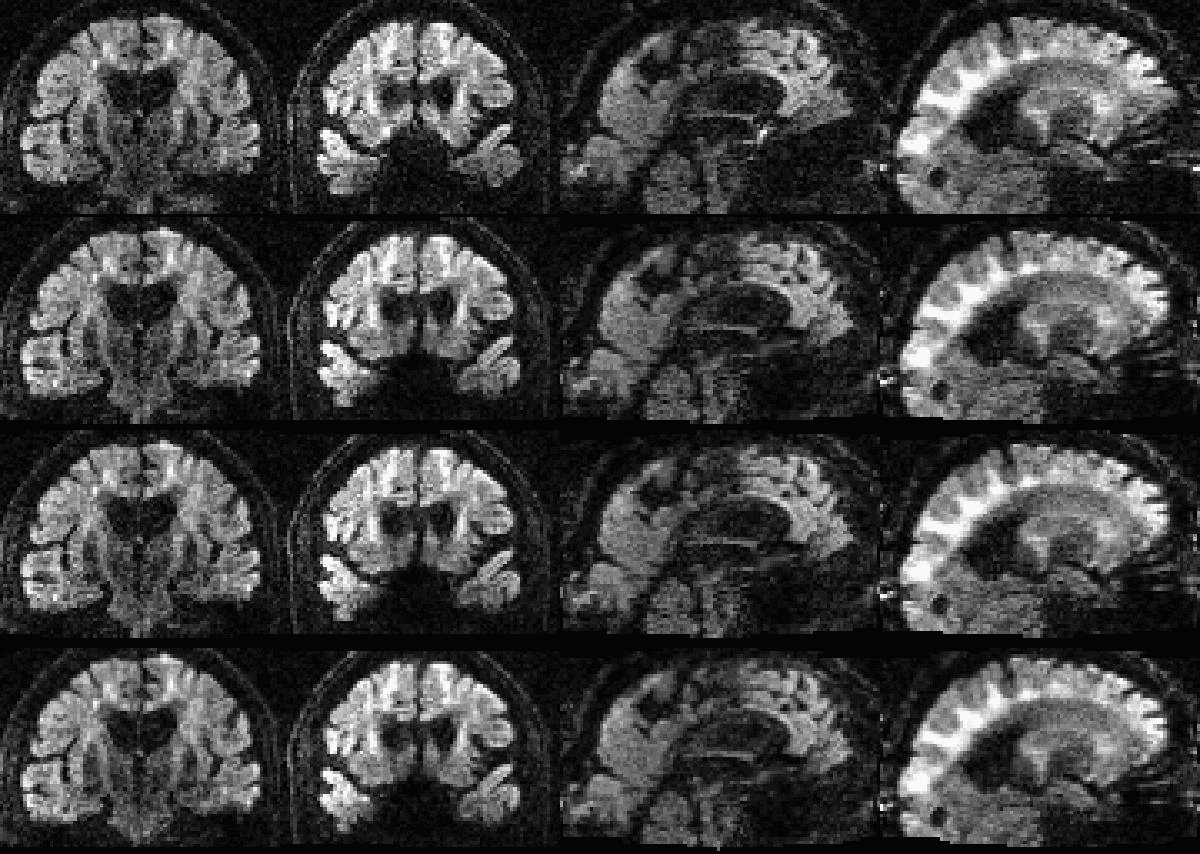

Supplement: Video 5 — The movie shows to coronal and two sagittal slices from each volume of a data set acquired on an elderly subject as part of the Whitehall study. The top row shows the original data. The second row shows the data after correction with the volume-to-volume model. The third row shows data after correction with the volume-to-volume model and outlier rejection and replacement (Andersson et al., 2016). The fourth row shows data after correction with the slice-to-volume model and outlier rejection and replacement. [file mmc5.gif]
