# Supplementary material for: Towards a comprehensive framework for movement and distortion correction of diffusion MR images: Within volume movement
Source: Neuroimage. 2017 May 15;152:450–66. doi: 10.1016/j.neuroimage.2017.02.085 (PMC5445723; doi:10.1016/j.neuroimage.2017.02.085)
Supplement: Application 1 [file mmc6.pdf]

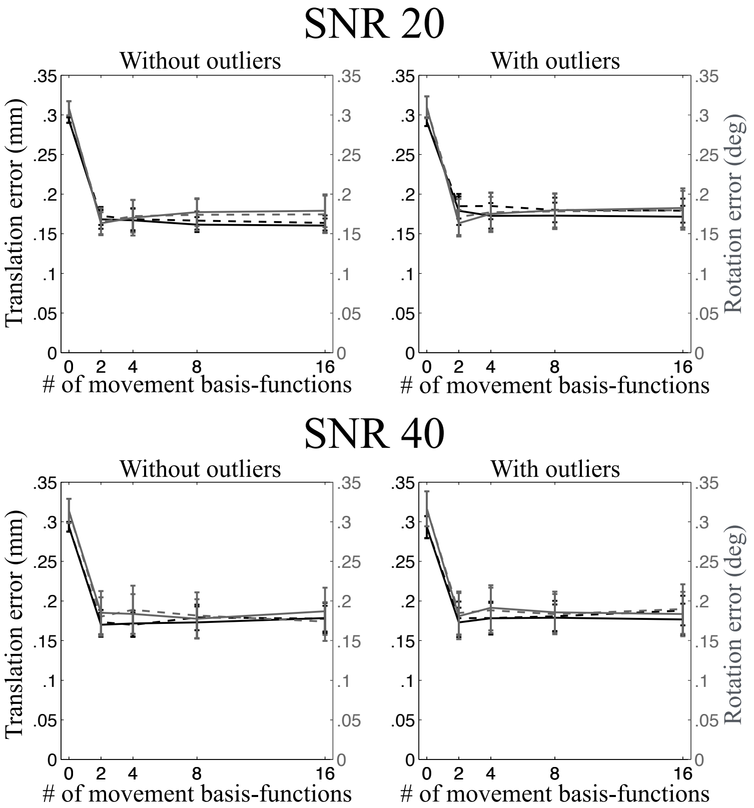

Figure S3: This figure shows the registration error for large movement and multi-band acquisition. The translation errors (averaged over all axes) are shown in black and the rotation errors (also averaged around all axes) are shown in grey. The solid lines pertain to regularisation of the movement with $\lambda = 1$ and the dashed lines with $\lambda = 10$.

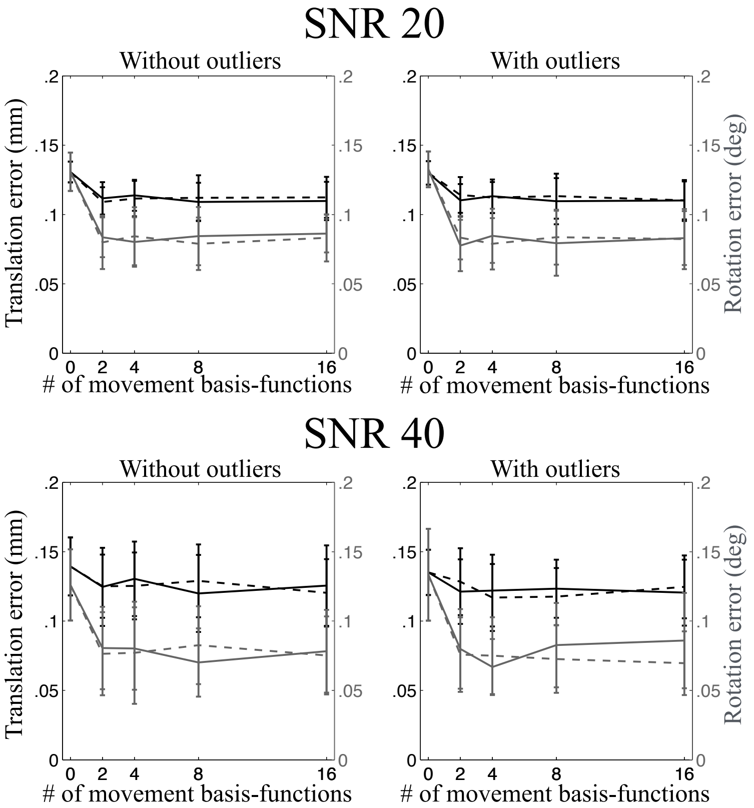

Figure S4: This figure shows the registration error for "normal" movement and multi-band acquisition. The translation errors (averaged over all axes) are shown in black and the rotation errors (also averaged around all axes) are shown in grey. The solid lines pertain to regularisation of the movement with $\lambda = 1$ and the dashed lines with $\lambda = 10$.

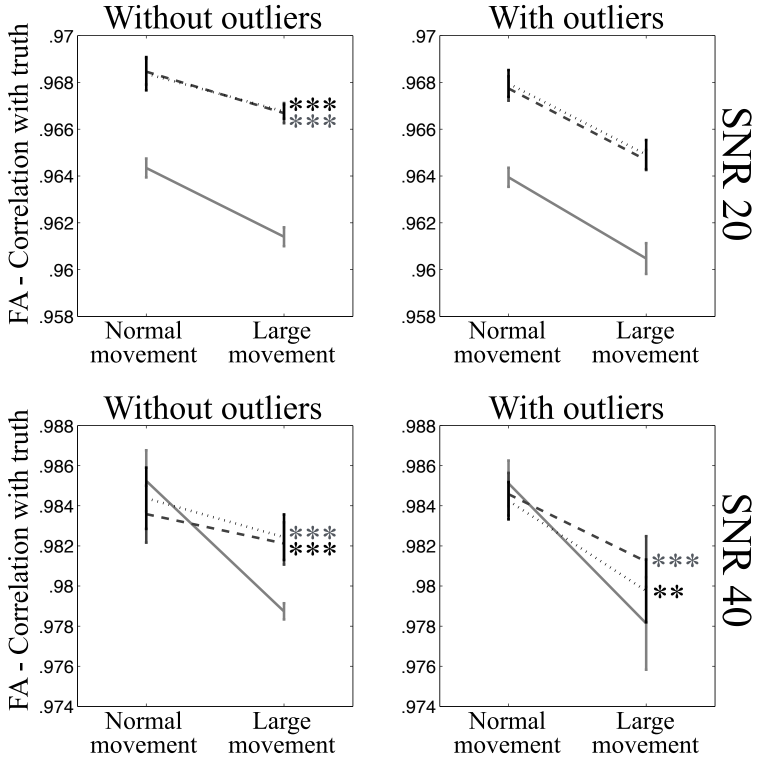

Figure S5: This figure shows the correlation between estimated and true FA for "normal" and "large" movement after correction of movements and distortions with `eddy` for the multi-band data with short TR. The solid line shows results after correction using the volume-to-volume model and the dashed and dotted lines using the slice-to-volume model with 8 and 16 basis-functions respectively. A statistical test (testing for unequal slopes) was performed to assess whether the difference between the "normal" and "large" movements was greater for volume-to-volume correction compared to the pertinent slice-to-volume model. Significance was indicated with * ($p \leq 0.05$), ** ($p \leq 0.01$) or *** ($p \leq 0.001$).

Band:		SB							
Movement:		Large							
Data:		Without outliers							
SNR:		40							
MP-order:		0	2		4		8		16	
λ:			1	10	1	10	1	10	1	10
b=0	xt:	1.130	0.430	0.430	0.261	0.259	0.157	0.154	0.179	0.154
	yt:	1.323	0.340	0.340	0.182	0.182	0.115	0.107	0.145	0.114
	zt:	1.081	0.353	0.353	0.202	0.202	0.111	0.110	0.116	0.106
	xr:	1.170	0.243	0.243	0.161	0.161	0.132	0.134	0.177	0.156
	yr:	0.582	0.461	0.462	0.315	0.314	0.227	0.224	0.226	0.226
	zr:	0.713	0.210	0.209	0.138	0.138	0.077	0.077	0.078	0.072
b=700	xt:	0.536	0.124	0.121	0.113	0.105	0.106	0.106	0.113	0.106
	yt:	0.490	0.236	0.240	0.290	0.255	0.285	0.255	0.256	0.231
	zt:	0.385	0.136	0.139	0.114	0.111	0.134	0.128	0.141	0.134
	xr:	0.459	0.088	0.095	0.081	0.091	0.091	0.094	0.106	0.086
	yr:	0.770	0.236	0.229	0.206	0.216	0.234	0.206	0.219	0.212
	zr:	0.470	0.106	0.117	0.107	0.118	0.133	0.132	0.119	0.123
b=2000	xt:	0.439	0.216	0.187	0.170	0.160	0.188	0.179	0.158	0.183
	yt:	0.369	0.278	0.286	0.321	0.305	0.301	0.289	0.294	0.273
	zt:	0.436	0.183	0.205	0.149	0.170	0.163	0.152	0.167	0.174
	xr:	0.543	0.123	0.155	0.133	0.142	0.153	0.149	0.141	0.138
	yr:	0.736	0.224	0.199	0.231	0.220	0.235	0.191	0.248	0.191
	zr:	0.269	0.127	0.129	0.107	0.129	0.157	0.152	0.130	0.147

Table S1: Registration error for single-band simulations with large motion, no outliers and an SNR of 40.

S.3 Tables of registration error

Band:		SB							
Movement:		Large							
Data:		Without outliers							
SNR:		20							
MP-order:		0	2		4		8		16	
λ:			1	10	1	10	1	10	1	10
b=0	xt:	1.130	0.430	0.430	0.261	0.259	0.165	0.157	0.181	0.157
	yt:	1.324	0.340	0.340	0.182	0.182	0.117	0.107	0.145	0.115
	zt:	1.081	0.353	0.353	0.202	0.202	0.111	0.111	0.117	0.107
	xr:	1.171	0.243	0.243	0.162	0.161	0.144	0.141	0.178	0.161
	yr:	0.583	0.461	0.462	0.315	0.314	0.224	0.224	0.227	0.225
	zr:	0.713	0.210	0.210	0.139	0.139	0.078	0.079	0.081	0.072
b=700	xt:	0.538	0.116	0.122	0.106	0.105	0.116	0.106	0.107	0.100
	yt:	0.471	0.226	0.241	0.271	0.228	0.246	0.227	0.278	0.209
	zt:	0.382	0.139	0.139	0.131	0.121	0.143	0.134	0.158	0.138
	xr:	0.455	0.091	0.092	0.097	0.098	0.105	0.105	0.127	0.097
	yr:	0.777	0.262	0.252	0.235	0.246	0.242	0.229	0.230	0.234
	zr:	0.472	0.123	0.117	0.127	0.118	0.107	0.120	0.136	0.116
b=2000	xt:	0.444	0.209	0.196	0.186	0.181	0.173	0.160	0.178	0.165
	yt:	0.368	0.265	0.268	0.286	0.268	0.282	0.260	0.281	0.260
	zt:	0.428	0.223	0.203	0.185	0.177	0.195	0.178	0.204	0.180
	xr:	0.533	0.143	0.143	0.161	0.142	0.201	0.165	0.204	0.168
	yr:	0.738	0.247	0.246	0.331	0.299	0.318	0.291	0.313	0.299
	zr:	0.266	0.120	0.113	0.114	0.124	0.128	0.124	0.129	0.116

Table S2: Registration error for single-band simulations with large motion, no outliers and an SNR of 20.

Band:		SB								
Movement:		Large								
Data:		With outliers								
SNR:		40								
MP-order:		0	2		4		8		16	
λ:			1	10	1	10	1	10	1	10
b=0	xt:	1.130	0.430	0.430	0.261	0.259	0.153	0.151	0.179	0.152
	yt:	1.323	0.340	0.340	0.182	0.182	0.114	0.107	0.145	0.113
	zt:	1.081	0.353	0.353	0.202	0.202	0.110	0.109	0.117	0.106
	xr:	1.170	0.243	0.243	0.161	0.161	0.126	0.128	0.177	0.150
	yr:	0.582	0.460	0.461	0.315	0.314	0.228	0.225	0.226	0.226
	zr:	0.713	0.209	0.209	0.138	0.138	0.078	0.078	0.077	0.075
b=700	xt:	0.537	0.122	0.126	0.117	0.113	0.123	0.108	0.120	0.113
	yt:	0.496	0.236	0.239	0.280	0.242	0.278	0.252	0.270	0.230
	zt:	0.383	0.146	0.132	0.143	0.139	0.161	0.141	0.160	0.145
	xr:	0.460	0.101	0.124	0.096	0.094	0.129	0.111	0.144	0.112
	yr:	0.772	0.225	0.224	0.210	0.216	0.243	0.230	0.234	0.225
	zr:	0.485	0.121	0.121	0.117	0.122	0.114	0.133	0.125	0.127
b=2000	xt:	0.441	0.184	0.223	0.198	0.169	0.156	0.183	0.169	0.195
	yt:	0.374	0.285	0.282	0.327	0.311	0.300	0.287	0.290	0.287
	zt:	0.437	0.190	0.197	0.174	0.159	0.168	0.170	0.169	0.178
	xr:	0.534	0.135	0.135	0.136	0.129	0.166	0.162	0.154	0.152
	yr:	0.738	0.206	0.205	0.222	0.232	0.256	0.213	0.249	0.215
	zr:	0.266	0.152	0.134	0.100	0.112	0.136	0.125	0.123	0.124

Table S3: Registration error for single-band simulations with large motion, outliers and an SNR of 40.

Band:		SB								
Movement:		Large								
Data:		With outliers								
SNR:		20								
MP-order:		0	2		4		8		16	
λ:			1	10	1	10	1	10	1	10
b=0	xt:	1.130	0.430	0.430	0.261	0.259	0.165	0.157	0.180	0.157
	yt:	1.324	0.340	0.340	0.182	0.182	0.118	0.107	0.145	0.114
	zt:	1.081	0.353	0.353	0.202	0.202	0.112	0.111	0.117	0.108
	xr:	1.171	0.243	0.243	0.162	0.162	0.144	0.141	0.176	0.161
	yr:	0.583	0.462	0.462	0.314	0.314	0.223	0.224	0.226	0.225
	zr:	0.713	0.210	0.210	0.139	0.139	0.079	0.079	0.076	0.072
b=700	xt:	0.539	0.122	0.124	0.106	0.102	0.117	0.108	0.125	0.112
	yt:	0.473	0.243	0.237	0.274	0.245	0.282	0.234	0.268	0.218
	zt:	0.385	0.147	0.149	0.136	0.136	0.166	0.150	0.175	0.149
	xr:	0.463	0.102	0.099	0.101	0.107	0.138	0.123	0.160	0.123
	yr:	0.767	0.270	0.253	0.253	0.254	0.257	0.241	0.256	0.236
	zr:	0.483	0.117	0.118	0.129	0.125	0.119	0.114	0.131	0.116
b=2000	xt:	0.444	0.201	0.201	0.179	0.191	0.176	0.172	0.186	0.171
	yt:	0.370	0.265	0.276	0.290	0.286	0.291	0.268	0.285	0.274
	zt:	0.429	0.209	0.216	0.193	0.197	0.206	0.217	0.195	0.190
	xr:	0.532	0.150	0.159	0.159	0.166	0.187	0.186	0.207	0.182
	yr:	0.737	0.270	0.266	0.331	0.302	0.333	0.299	0.332	0.285
	zr:	0.266	0.132	0.121	0.117	0.118	0.128	0.129	0.135	0.116

Table S4: Registration error for single-band simulations with large motion, outliers and an SNR of 20.

Band:		SB								
Movement:		Normal								
Data:		Without outliers								
SNR:		40								
MP-order:		0	2		4		8		16	
λ:			1	10	1	10	1	10	1	10
b=0	xt:	0.377	0.142	0.142	0.082	0.082	0.050	0.042	0.052	0.050
	yt:	0.444	0.121	0.121	0.075	0.075	0.060	0.060	0.065	0.067
	zt:	0.359	0.125	0.125	0.076	0.075	0.074	0.067	0.066	0.066
	xr:	0.404	0.083	0.083	0.059	0.059	0.063	0.062	0.082	0.077
	yr:	0.200	0.135	0.135	0.072	0.072	0.046	0.046	0.056	0.049
	zr:	0.236	0.069	0.069	0.047	0.047	0.028	0.026	0.057	0.046
b=700	xt:	0.245	0.086	0.078	0.082	0.102	0.093	0.113	0.115	0.111
	yt:	0.273	0.121	0.125	0.147	0.133	0.146	0.145	0.167	0.137
	zt:	0.132	0.097	0.104	0.105	0.103	0.105	0.082	0.088	0.088
	xr:	0.169	0.039	0.043	0.049	0.043	0.063	0.065	0.077	0.062
	yr:	0.294	0.138	0.138	0.122	0.124	0.157	0.153	0.183	0.188
	zr:	0.154	0.035	0.034	0.060	0.038	0.050	0.051	0.060	0.048
b=2000	xt:	0.200	0.249	0.248	0.238	0.208	0.241	0.275	0.228	0.193
	yt:	0.246	0.261	0.251	0.271	0.258	0.263	0.262	0.266	0.262
	zt:	0.197	0.169	0.166	0.168	0.164	0.194	0.157	0.154	0.150
	xr:	0.197	0.087	0.073	0.090	0.094	0.102	0.098	0.113	0.100
	yr:	0.257	0.099	0.101	0.109	0.154	0.165	0.156	0.148	0.166
	zr:	0.189	0.123	0.137	0.180	0.150	0.180	0.161	0.182	0.135

Table S5: Registration error for single-band simulations with normal motion, no outliers and an SNR of 40.

Band:		SB							
Movement:		Normal							
Data:		Without outliers							
SNR:		20							
MP-order:		0	2		4		8		16	
λ:			1	10	1	10	1	10	1	10
b=0	xt:	0.377	0.141	0.142	0.081	0.080	0.051	0.047	0.047	0.049
	yt:	0.444	0.119	0.120	0.073	0.074	0.061	0.062	0.064	0.068
	zt:	0.359	0.130	0.126	0.076	0.077	0.075	0.069	0.069	0.065
	xr:	0.404	0.082	0.083	0.059	0.060	0.064	0.062	0.079	0.078
	yr:	0.200	0.138	0.137	0.073	0.073	0.046	0.045	0.054	0.049
	zr:	0.236	0.070	0.070	0.047	0.047	0.029	0.028	0.059	0.046
b=700	xt:	0.210	0.095	0.093	0.094	0.112	0.111	0.087	0.111	0.106
	yt:	0.205	0.155	0.137	0.151	0.135	0.172	0.133	0.171	0.150
	zt:	0.144	0.096	0.117	0.116	0.105	0.125	0.128	0.100	0.116
	xr:	0.154	0.055	0.044	0.048	0.055	0.060	0.061	0.076	0.058
	yr:	0.341	0.184	0.148	0.157	0.174	0.184	0.143	0.199	0.170
	zr:	0.150	0.040	0.050	0.076	0.058	0.058	0.089	0.077	0.085
b=2000	xt:	0.209	0.152	0.139	0.164	0.161	0.157	0.187	0.161	0.151
	yt:	0.251	0.232	0.235	0.240	0.237	0.247	0.233	0.256	0.234
	zt:	0.179	0.147	0.163	0.183	0.164	0.188	0.191	0.135	0.162
	xr:	0.199	0.098	0.099	0.116	0.112	0.130	0.123	0.145	0.122
	yr:	0.253	0.219	0.212	0.217	0.236	0.243	0.208	0.286	0.250
	zr:	0.213	0.108	0.090	0.103	0.097	0.106	0.109	0.112	0.100

Table S6: Registration error for single-band simulations with normal motion, no outliers and an SNR of 20.

Band:		SB								
Movement:		Normal								
Data:		With outliers								
SNR:		40								
MP-order:		0	2		4		8		16	
λ:			1	10	1	10	1	10	1	10
b=0	xt:	0.377	0.142	0.142	0.083	0.081	0.051	0.040	0.052	0.053
	yt:	0.444	0.121	0.121	0.075	0.074	0.060	0.060	0.065	0.069
	zt:	0.359	0.125	0.125	0.076	0.076	0.075	0.066	0.067	0.068
	xr:	0.404	0.083	0.083	0.059	0.059	0.063	0.062	0.082	0.079
	yr:	0.199	0.135	0.136	0.072	0.072	0.045	0.046	0.055	0.049
	zr:	0.236	0.069	0.069	0.047	0.047	0.028	0.025	0.056	0.044
b=700	xt:	0.236	0.074	0.065	0.080	0.093	0.084	0.104	0.099	0.085
	yt:	0.251	0.148	0.148	0.187	0.150	0.164	0.157	0.173	0.131
	zt:	0.150	0.130	0.142	0.152	0.135	0.170	0.125	0.150	0.144
	xr:	0.158	0.067	0.061	0.079	0.071	0.096	0.100	0.122	0.091
	yr:	0.316	0.135	0.196	0.158	0.175	0.154	0.208	0.192	0.161
	zr:	0.154	0.041	0.053	0.069	0.067	0.071	0.074	0.082	0.061
b=2000	xt:	0.217	0.207	0.214	0.205	0.187	0.248	0.240	0.231	0.210
	yt:	0.251	0.255	0.258	0.265	0.254	0.278	0.261	0.269	0.250
	zt:	0.211	0.172	0.180	0.181	0.173	0.196	0.144	0.161	0.167
	xr:	0.199	0.105	0.092	0.121	0.108	0.130	0.125	0.157	0.127
	yr:	0.257	0.124	0.115	0.146	0.158	0.179	0.157	0.199	0.150
	zr:	0.175	0.114	0.131	0.114	0.095	0.138	0.138	0.158	0.120

Table S7: Registration error for single-band simulations with normal motion, outliers and an SNR of 40.

Band:		SB							
Movement:		Normal							
Data:		With outliers							
SNR:		20							
MP-order:		0	2		4		8		16
λ:			1	10	1	10	1	10	1	10
b=0	xt:	0.377	0.141	0.141	0.081	0.081	0.052	0.048	0.048	0.050
	yt:	0.444	0.119	0.119	0.074	0.074	0.061	0.063	0.065	0.068
	zt:	0.359	0.130	0.130	0.075	0.076	0.075	0.067	0.068	0.065
	xr:	0.404	0.082	0.082	0.059	0.059	0.063	0.062	0.080	0.078
	yr:	0.200	0.138	0.138	0.072	0.073	0.046	0.044	0.055	0.049
	zr:	0.236	0.070	0.070	0.047	0.047	0.029	0.028	0.057	0.046
b=700	xt:	0.207	0.091	0.089	0.102	0.081	0.096	0.091	0.104	0.099
	yt:	0.206	0.134	0.132	0.178	0.154	0.177	0.142	0.168	0.143
	zt:	0.151	0.138	0.131	0.156	0.151	0.178	0.166	0.157	0.162
	xr:	0.153	0.066	0.067	0.080	0.077	0.096	0.090	0.124	0.095
	yr:	0.365	0.196	0.234	0.203	0.169	0.180	0.174	0.228	0.165
	zr:	0.153	0.076	0.046	0.069	0.058	0.078	0.080	0.091	0.088
b=2000	xt:	0.206	0.156	0.137	0.156	0.161	0.158	0.168	0.179	0.169
	yt:	0.253	0.244	0.246	0.255	0.242	0.253	0.246	0.260	0.245
	zt:	0.181	0.182	0.165	0.174	0.176	0.188	0.183	0.163	0.167
	xr:	0.192	0.111	0.115	0.134	0.133	0.159	0.144	0.193	0.148
	yr:	0.256	0.213	0.228	0.270	0.236	0.266	0.242	0.277	0.249
	zr:	0.218	0.101	0.107	0.094	0.111	0.113	0.110	0.121	0.104

Table S8: Registration error for single-band simulations with normal motion, outliers and an SNR of 20.

Band:		MB3								
Movement:		Large								
Data:		Without outliers								
SNR:		40								
MP-order:		0	2		4		8		16	
λ:			1	10	1	10	1	10	1	10
b=0	xt:	0.222	0.089	0.089	0.087	0.087	0.087	0.087	0.086	0.087
	yt:	0.187	0.080	0.080	0.079	0.079	0.078	0.079	0.078	0.078
	zt:	0.103	0.048	0.047	0.051	0.050	0.047	0.048	0.048	0.045
	xr:	0.212	0.079	0.079	0.078	0.078	0.078	0.079	0.081	0.078
	yr:	0.351	0.177	0.177	0.172	0.172	0.177	0.174	0.176	0.178
	zr:	0.182	0.105	0.105	0.103	0.103	0.104	0.104	0.105	0.105
b=700	xt:	0.370	0.120	0.121	0.127	0.124	0.123	0.119	0.123	0.123
	yt:	0.217	0.177	0.172	0.189	0.196	0.211	0.182	0.191	0.190
	zt:	0.300	0.127	0.129	0.136	0.130	0.131	0.128	0.131	0.127
	xr:	0.380	0.130	0.139	0.126	0.119	0.135	0.115	0.124	0.138
	yr:	0.620	0.385	0.354	0.376	0.396	0.395	0.416	0.415	0.384
	zr:	0.379	0.152	0.145	0.169	0.168	0.180	0.177	0.174	0.174
b=2000	xt:	0.402	0.209	0.215	0.201	0.183	0.188	0.201	0.191	0.203
	yt:	0.429	0.295	0.293	0.299	0.299	0.293	0.296	0.290	0.304
	zt:	0.327	0.136	0.149	0.133	0.149	0.131	0.141	0.122	0.136
	xr:	0.209	0.080	0.073	0.103	0.088	0.092	0.069	0.084	0.098
	yr:	0.256	0.162	0.133	0.165	0.150	0.127	0.180	0.179	0.165
	zr:	0.198	0.127	0.129	0.157	0.120	0.126	0.137	0.126	0.150

Table S9: Registration error for single-band simulations with long TR, large motion, no outliers and an SNR of 40.

Band:		MB3								
Movement:		Large								
Data:		Without outliers								
SNR:		20								
MP-order:		0	2		4		8		16	
λ:			1	10	1	10	1	10	1	10
b=0	xt:	0.222	0.089	0.089	0.087	0.087	0.087	0.087	0.086	0.087
	yt:	0.188	0.080	0.080	0.079	0.079	0.078	0.079	0.078	0.079
	zt:	0.103	0.047	0.048	0.048	0.048	0.049	0.049	0.052	0.049
	xr:	0.212	0.079	0.079	0.078	0.078	0.079	0.079	0.081	0.079
	yr:	0.351	0.176	0.176	0.174	0.175	0.174	0.173	0.172	0.171
	zr:	0.182	0.105	0.105	0.104	0.104	0.104	0.103	0.104	0.103
b=700	xt:	0.370	0.126	0.124	0.121	0.129	0.123	0.134	0.124	0.122
	yt:	0.216	0.156	0.153	0.151	0.152	0.153	0.154	0.161	0.154
	zt:	0.305	0.145	0.149	0.145	0.141	0.142	0.143	0.145	0.144
	xr:	0.376	0.109	0.115	0.110	0.120	0.114	0.123	0.121	0.112
	yr:	0.615	0.411	0.398	0.380	0.382	0.393	0.364	0.388	0.390
	zr:	0.371	0.160	0.185	0.165	0.153	0.186	0.158	0.159	0.150
b=2000	xt:	0.407	0.179	0.196	0.167	0.190	0.165	0.198	0.168	0.175
	yt:	0.424	0.265	0.281	0.272	0.267	0.273	0.282	0.264	0.270
	zt:	0.316	0.147	0.169	0.159	0.160	0.161	0.175	0.163	0.172
	xr:	0.202	0.095	0.079	0.093	0.086	0.094	0.085	0.099	0.091
	yr:	0.253	0.173	0.172	0.174	0.166	0.182	0.170	0.166	0.169
	zr:	0.196	0.094	0.096	0.078	0.077	0.079	0.086	0.091	0.084

Table S10: Registration error for single-band simulations with long TR, large motion, no outliers and an SNR of 20.

Band:		MB3 Short TR								
Movement:		Large								
Data:		With outliers								
SNR:		40								
MP-order:		0	2		4		8		16	
λ:			1	10	1	10	1	10	1	10
b=0	xt:	0.221	0.090	0.090	0.088	0.088	0.088	0.088	0.088	0.088
	yt:	0.190	0.080	0.080	0.079	0.079	0.079	0.079	0.079	0.079
	zt:	0.105	0.054	0.055	0.055	0.054	0.061	0.060	0.061	0.060
	xr:	0.210	0.085	0.085	0.083	0.083	0.086	0.085	0.088	0.086
	yr:	0.349	0.179	0.179	0.177	0.178	0.172	0.171	0.171	0.171
	zr:	0.178	0.104	0.104	0.103	0.103	0.102	0.101	0.102	0.101
b=700	xt:	0.380	0.201	0.200	0.206	0.203	0.208	0.205	0.205	0.202
	yt:	0.246	0.210	0.213	0.223	0.221	0.227	0.207	0.212	0.219
	zt:	0.313	0.206	0.207	0.208	0.206	0.209	0.207	0.211	0.208
	xr:	0.382	0.186	0.194	0.189	0.188	0.197	0.186	0.204	0.184
	yr:	0.626	0.460	0.460	0.475	0.475	0.446	0.455	0.452	0.459
	zr:	0.373	0.182	0.185	0.190	0.186	0.197	0.197	0.190	0.192
b=2000	xt:	0.410	0.224	0.232	0.244	0.228	0.231	0.233	0.228	0.276
	yt:	0.444	0.326	0.337	0.330	0.342	0.335	0.342	0.330	0.354
	zt:	0.328	0.166	0.193	0.172	0.185	0.172	0.207	0.177	0.208
	xr:	0.244	0.119	0.122	0.124	0.126	0.135	0.129	0.131	0.128
	yr:	0.276	0.199	0.180	0.213	0.197	0.176	0.159	0.183	0.187
	zr:	0.208	0.115	0.154	0.169	0.158	0.160	0.166	0.132	0.200

Table S11: Registration error for single-band simulations with long TR, large motion, outliers and an SNR of 40.

Band:		MB3 Short TR								
Movement:		Large								
Data:		With outliers								
SNR:		20								
MP-order:		0	2		4		8		16	
λ:			1	10	1	10	1	10	1	10
b=0	xt:	0.221	0.089	0.089	0.088	0.087	0.088	0.088	0.087	0.088
	yt:	0.190	0.081	0.081	0.079	0.080	0.079	0.079	0.079	0.079
	zt:	0.105	0.072	0.072	0.064	0.068	0.059	0.058	0.061	0.058
	xr:	0.210	0.090	0.090	0.086	0.087	0.085	0.085	0.088	0.086
	yr:	0.349	0.169	0.169	0.172	0.170	0.174	0.174	0.172	0.173
	zr:	0.178	0.102	0.102	0.101	0.101	0.102	0.102	0.102	0.102
b=700	xt:	0.386	0.206	0.206	0.208	0.209	0.209	0.205	0.208	0.205
	yt:	0.251	0.196	0.195	0.194	0.201	0.202	0.196	0.197	0.197
	zt:	0.315	0.220	0.221	0.216	0.224	0.217	0.215	0.218	0.213
	xr:	0.379	0.174	0.176	0.192	0.191	0.196	0.184	0.204	0.190
	yr:	0.593	0.397	0.385	0.418	0.420	0.438	0.425	0.439	0.449
	zr:	0.370	0.171	0.188	0.179	0.198	0.187	0.190	0.209	0.190
b=2000	xt:	0.408	0.226	0.241	0.212	0.240	0.210	0.242	0.206	0.233
	yt:	0.427	0.292	0.315	0.292	0.316	0.303	0.324	0.298	0.319
	zt:	0.318	0.229	0.244	0.199	0.241	0.190	0.214	0.189	0.222
	xr:	0.250	0.120	0.135	0.144	0.147	0.142	0.144	0.139	0.141
	yr:	0.248	0.152	0.175	0.183	0.164	0.190	0.189	0.190	0.175
	zr:	0.209	0.096	0.121	0.100	0.115	0.101	0.110	0.099	0.109

Table S12: Registration error for single-band simulations with long TR, large motion, outliers and an SNR of 20.

Band:		MB3 Short TR								
Movement:		Normal								
Data:		Without outliers								
SNR:		40								
MP-order:		0	2		4		8		16	
λ:			1	10	1	10	1	10	1	10
b=0	xt:	0.072	0.023	0.023	0.026	0.024	0.034	0.029	0.036	0.031
	yt:	0.062	0.027	0.026	0.025	0.025	0.024	0.024	0.024	0.024
	zt:	0.043	0.035	0.035	0.035	0.035	0.036	0.035	0.036	0.035
	xr:	0.075	0.036	0.037	0.040	0.039	0.043	0.042	0.045	0.042
	yr:	0.107	0.038	0.038	0.038	0.038	0.040	0.040	0.041	0.040
	zr:	0.052	0.015	0.015	0.013	0.013	0.015	0.014	0.016	0.014
b=700	xt:	0.163	0.128	0.150	0.133	0.120	0.119	0.118	0.121	0.104
	yt:	0.133	0.207	0.185	0.244	0.201	0.187	0.242	0.215	0.198
	zt:	0.139	0.112	0.101	0.110	0.118	0.127	0.108	0.114	0.110
	xr:	0.133	0.057	0.053	0.047	0.062	0.063	0.043	0.058	0.056
	yr:	0.235	0.151	0.120	0.189	0.133	0.097	0.152	0.159	0.171
	zr:	0.134	0.042	0.046	0.045	0.039	0.040	0.037	0.047	0.042
b=2000	xt:	0.209	0.194	0.215	0.203	0.196	0.161	0.192	0.182	0.186
	yt:	0.287	0.293	0.292	0.290	0.297	0.285	0.301	0.296	0.288
	zt:	0.146	0.104	0.099	0.108	0.112	0.106	0.111	0.105	0.108
	xr:	0.122	0.085	0.086	0.091	0.088	0.084	0.077	0.094	0.076
	yr:	0.135	0.119	0.105	0.104	0.125	0.105	0.113	0.115	0.101
	zr:	0.142	0.182	0.188	0.154	0.157	0.143	0.227	0.129	0.135

Table S13: Registration error for single-band simulations with long TR, normal motion, no outliers and an SNR of 40.

Band:		MB3 Short TR								
Movement:		Normal								
Data:		Without outliers								
SNR:		20								
MP-order:		0	2		4		8		16	
λ:			1	10	1	10	1	10	1	10
b=0	xt:	0.072	0.052	0.052	0.052	0.052	0.030	0.033	0.033	0.024
	yt:	0.062	0.022	0.022	0.023	0.023	0.024	0.025	0.024	0.025
	zt:	0.043	0.044	0.044	0.044	0.044	0.036	0.039	0.036	0.035
	xr:	0.075	0.054	0.054	0.056	0.057	0.043	0.047	0.045	0.041
	yr:	0.108	0.048	0.048	0.049	0.049	0.040	0.044	0.041	0.039
	zr:	0.052	0.022	0.022	0.022	0.022	0.014	0.018	0.016	0.014
b=700	xt:	0.166	0.099	0.095	0.102	0.094	0.119	0.130	0.124	0.127
	yt:	0.138	0.167	0.161	0.173	0.157	0.155	0.150	0.161	0.168
	zt:	0.124	0.131	0.137	0.136	0.136	0.119	0.125	0.119	0.114
	xr:	0.142	0.052	0.046	0.058	0.048	0.054	0.052	0.054	0.054
	yr:	0.292	0.191	0.177	0.140	0.171	0.180	0.181	0.204	0.193
	zr:	0.124	0.040	0.037	0.043	0.046	0.064	0.052	0.059	0.058
b=2000	xt:	0.173	0.102	0.090	0.101	0.117	0.120	0.129	0.133	0.142
	yt:	0.266	0.268	0.256	0.269	0.259	0.266	0.262	0.250	0.257
	zt:	0.133	0.121	0.125	0.124	0.122	0.111	0.115	0.109	0.119
	xr:	0.136	0.076	0.086	0.093	0.100	0.103	0.085	0.095	0.084
	yr:	0.125	0.113	0.111	0.117	0.112	0.131	0.118	0.126	0.116
	zr:	0.125	0.156	0.140	0.144	0.155	0.131	0.114	0.137	0.150

Table S14: Registration error for single-band simulations with long TR, normal motion, no outliers and an SNR of 20.

Band:		MB3 Short TR								
Movement:		Normal								
Data:		With outliers								
SNR:		40								
MP-order:		0	2		4		8		16	
λ:			1	10	1	10	1	10	1	10
b=0	xt:	0.072	0.024	0.024	0.027	0.025	0.034	0.030	0.036	0.031
	yt:	0.062	0.026	0.026	0.025	0.025	0.024	0.024	0.024	0.024
	zt:	0.043	0.035	0.035	0.035	0.035	0.036	0.035	0.036	0.035
	xr:	0.074	0.038	0.038	0.039	0.039	0.043	0.042	0.045	0.042
	yr:	0.108	0.038	0.038	0.039	0.038	0.040	0.040	0.041	0.040
	zr:	0.052	0.014	0.014	0.013	0.013	0.015	0.014	0.016	0.014
b=700	xt:	0.169	0.144	0.127	0.120	0.124	0.116	0.123	0.112	0.118
	yt:	0.155	0.176	0.201	0.182	0.167	0.185	0.158	0.185	0.191
	zt:	0.120	0.109	0.119	0.115	0.109	0.116	0.114	0.128	0.130
	xr:	0.144	0.062	0.056	0.049	0.054	0.058	0.066	0.063	0.054
	yr:	0.263	0.166	0.124	0.109	0.158	0.157	0.133	0.129	0.098
	zr:	0.125	0.053	0.049	0.054	0.043	0.049	0.048	0.055	0.061
b=2000	xt:	0.186	0.180	0.210	0.177	0.167	0.178	0.158	0.158	0.187
	yt:	0.267	0.291	0.304	0.305	0.293	0.303	0.303	0.292	0.287
	zt:	0.141	0.107	0.110	0.112	0.108	0.118	0.115	0.112	0.119
	xr:	0.175	0.091	0.071	0.079	0.089	0.094	0.089	0.113	0.072
	yr:	0.136	0.101	0.106	0.105	0.105	0.114	0.102	0.106	0.118
	zr:	0.123	0.156	0.186	0.114	0.135	0.172	0.120	0.205	0.127

Table S15: Registration error for single-band simulations with long TR, normal motion, outliers and an SNR of 40.

Band:		MB3 Short TR								
Movement:		Normal								
Data:		With outliers								
SNR:		20								
MP-order:		0	2		4		8		16	
λ:			1	10	1	10	1	10	1	10
b=0	xt:	0.072	0.052	0.052	0.053	0.052	0.030	0.033	0.033	0.025
	yt:	0.062	0.022	0.022	0.023	0.023	0.025	0.026	0.024	0.025
	zt:	0.043	0.044	0.044	0.044	0.044	0.036	0.040	0.036	0.035
	xr:	0.075	0.054	0.054	0.056	0.057	0.043	0.047	0.045	0.041
	yr:	0.108	0.048	0.048	0.049	0.049	0.040	0.044	0.041	0.039
	zr:	0.052	0.021	0.021	0.021	0.021	0.014	0.018	0.016	0.014
b=700	xt:	0.166	0.085	0.098	0.113	0.096	0.104	0.121	0.101	0.124
	yt:	0.144	0.165	0.174	0.162	0.162	0.167	0.150	0.158	0.156
	zt:	0.120	0.124	0.130	0.135	0.138	0.128	0.118	0.126	0.119
	xr:	0.138	0.050	0.051	0.056	0.052	0.070	0.059	0.060	0.070
	yr:	0.290	0.164	0.172	0.149	0.156	0.156	0.199	0.189	0.172
	zr:	0.124	0.057	0.050	0.053	0.043	0.062	0.049	0.069	0.055
b=2000	xt:	0.171	0.104	0.110	0.097	0.111	0.131	0.151	0.139	0.132
	yt:	0.264	0.272	0.271	0.266	0.265	0.260	0.265	0.259	0.264
	zt:	0.127	0.124	0.125	0.125	0.119	0.106	0.116	0.115	0.112
	xr:	0.145	0.081	0.083	0.100	0.084	0.103	0.086	0.099	0.089
	yr:	0.122	0.108	0.117	0.117	0.117	0.132	0.117	0.123	0.133
	zr:	0.140	0.116	0.153	0.161	0.131	0.093	0.134	0.106	0.126

Table S16: Registration error for single-band simulations with long TR, normal motion, outliers and an SNR of 20.
